# Reverse AD at Higher Types: Pure, Principled and Denotationally Correct

**DOI:** 10.1007/978-3-030-72019-3_22

**Published:** 2021-03-23

**Authors:** Matthijs Vákár

**Affiliations:** Imperial College, London, UK; Utrecht University, Utrecht, Netherlands

**Keywords:** automatic differentiation, program correctness, semantics

## Abstract

We show how to define forward- and reverse-mode automatic differentiation source-code transformations or on a standard higher-order functional language. The transformations generate purely functional code, and they are principled in the sense that their definition arises from a categorical universal property. We give a semantic proof of correctness of the transformations. In their most elegant formulation, the transformations generate code with linear types. However, we demonstrate how the transformations can be implemented in a standard functional language without sacrificing correctness. To do so, we make use of abstract data types to represent the required linear types, e.g. through the use of a basic module system.

### 1 Introduction

Automatic differentiation (AD) is a technique for transforming code that implements a function $f$ into code that computes $f$'s derivative, essentially by using the chain rule for derivatives. Due to its efficiency and numerical stability, AD is the technique of choice whenever derivatives need to be computed of functions that are implemented as programs, particularly in high dimensional settings. Optimization and Monte-Carlo integration algorithms, such as gradient descent and Hamiltonian Monte-Carlo methods, rely crucially on the calculation of derivatives. These algorithms are used in virtually every machine learning and computational statistics application, and the calculation of derivatives is usually the computational bottleneck. These applications explain the recent surge of interest in AD, which has resulted in the proliferation of popular AD systems such as TensorFlow [1], PyTorch [30], and Stan Math [9].

AD, roughly speaking, comes in two modes: forward-mode and reverse-mode. When differentiating a function $\mathbb{R}^n \to \mathbb{R}^m$, forward-mode tends to be more efficient if $m \gg n$, while reverse-mode generally is more performant if $n \gg m$. As most applications reduce to optimization or Monte-Carlo integration of an objective function $\mathbb{R}^n \to \mathbb{R}$ with $n$ very large (today, in the order of $10^4 - 10^7$), reverse-mode AD is in many ways the more interesting algorithm.

However, reverse AD is also more complicated to understand and implement than forward AD. Forward AD can be implemented as a structure-preserving program transformation, even on languages with complex features [32]. As such, it admits an elegant proof of correctness [20]. By contrast, reverse-AD is only well-understood as a source-code transformation (also called *define-then-run* style AD) on limited programming languages. Typically, its implementations on more expressive languages that have features such as higher-order functions make use of *define-by-run* approaches. These approaches first build a computation graph during runtime, effectively evaluating the program until a straight-line first-order program is left, and then they evaluate this new program [30,9]. Such approaches have the severe downside that the differentiated code cannot benefit from existing optimizing compiler architectures. As such, these AD libraries need to be implemented using carefully, manually optimized code, that for example does not contain any common subexpressions. This implementation process is precarious and labour intensive. Further, some whole-program optimizations that a compiler would detect go entirely unused in such systems.

Similarly, correctness proofs of reverse AD have taken a define-by-run approach and have relied on non-standard operational semantics, using forms of symbolic execution [2,28,8]. Most work that treats reverse-AD as a source-code transformation does so by making use of complex transformations which introduce mutable state and/or non-local control flow [31,38]. As a result, we are not sure whether and why such techniques are correct. Another approach has been to compile high-level languages to a low-level imperative representation first, and then to perform AD at that level [22], using mutation and jumps. This approach has the downside that we might lose important opportunities for compiler optimizations, such as map-fusion and embarrassingly parallel maps, which we can exploit if we perform define-then-run AD on a high-level representation.

A notable exception to these define-by-run and non-functional approaches to AD is [16], which presents an elegant, purely functional, define-then-run version of reverse AD. Unfortunately, their techniques are limited to first-order programs over tuples of real numbers. This paper extends the work of [16] to apply to higher-order programs over (primitive) arrays of reals:

- It defines purely functional define-then-run reverse-mode AD on a higher-order language.
- It shows how the resulting, mysterious looking program transformation arises from a universal property if we phrase the problem in a suitable categorical language. Consequently, the transformations automatically respect equational reasoning principles.
- It explains, from this categorical setting, precisely in what sense reverse AD is the "mirror image" of forward AD.
- It presents an elegant proof of semantic correctness of the AD transformations, based on a semantic logical relations argument, demonstrating that the transformations calculate the derivatives of the program in the usual mathematical sense.
- It shows that the AD definitions and correctness proof are extensible to higher-order primitives such as a **map**-operation over our primitive arrays.
- It discusses how our techniques are readily implementable in standard functional languages to give purely functional, principled, semantically correct, define-then-run reverse-mode AD.

### 2 Key Ideas

Consider a simple programming language. Types are statically sized arrays $\mathbf{real}^n$ for some $n$, and programs are obtained from a collection of (unary) primitive operations $x : \mathbf{real}^n \vdash \mathsf{op}(x) : \mathbf{real}^m$ (intended to implement differentiable functions like linear algebra operations and sigmoid functions) by sequencing.

We can implement both forward mode $\overrightarrow{\mathcal{D}}$ and reverse mode AD $\overleftarrow{\mathcal{D}}$ on this language as source-code translations to the larger language of a simply typed $\lambda$-calculus over the ground types $\mathbf{real}^n$ that includes at least the same operations. Forward (resp. reverse) AD translates a type $\tau$ to a pair of types $\overrightarrow{\mathcal{D}}(\tau) = (\overrightarrow{\mathcal{D}}(\tau)_1, \overrightarrow{\mathcal{D}}(\tau)_2)$ (resp. $\overleftarrow{\mathcal{D}}(\tau) = (\overleftarrow{\mathcal{D}}(\tau)_1, \overleftarrow{\mathcal{D}}(\tau)_2)$) – the first component for holding function values, also called *primals* in the AD literature; the second component for holding derivative values, also called *tangents* (resp. *adjoints* or *cotangents*):

$$\overrightarrow{\mathcal{D}}(\mathbf{real}^n) \stackrel{\text{def}}{=} \overleftarrow{\mathcal{D}}(\mathbf{real}^n) = (\mathbf{real}^n, \mathbf{real}^n).$$

We translate terms $x : \tau \vdash t : \sigma$ to pairs of terms $\overrightarrow{\mathcal{D}}(t) = (\overrightarrow{\mathcal{D}}(t)_1, \overrightarrow{\mathcal{D}}(t)_2)$ for forward AD and $\overleftarrow{\mathcal{D}}(t) = (\overleftarrow{\mathcal{D}}(t)_1, \overleftarrow{\mathcal{D}}(t)_2)$ for reverse AD, which have types

$$\begin{array}{lcl} x : \overrightarrow{\mathcal{D}}(\tau)_1 \vdash \overrightarrow{\mathcal{D}}(t)_1 : \overrightarrow{\mathcal{D}}(\sigma)_1 & \text{and} & x : \overleftarrow{\mathcal{D}}(\tau)_1 \vdash \overleftarrow{\mathcal{D}}(t)_1 : \overleftarrow{\mathcal{D}}(\sigma)_1 \\ x : \overrightarrow{\mathcal{D}}(\tau)_1 \vdash \overrightarrow{\mathcal{D}}(t)_2 : \overrightarrow{\mathcal{D}}(\tau)_2 \to \overrightarrow{\mathcal{D}}(\sigma)_2 & & x : \overleftarrow{\mathcal{D}}(\tau)_1 \vdash \overleftarrow{\mathcal{D}}(t)_2 : \overleftarrow{\mathcal{D}}(\sigma)_2 \to \overleftarrow{\mathcal{D}}(\tau)_2. \end{array}$$

$\overrightarrow{\mathcal{D}}(t)_1$ and $\overleftarrow{\mathcal{D}}(t)_1$ perform the primal computations for the program $t$, while $\overrightarrow{\mathcal{D}}(t)_2$ and $\overleftarrow{\mathcal{D}}(t)_2$ compute the derivatives, resp., for forward and reverse AD.

Indeed, we define, by induction on the syntax:

$$\overrightarrow{\mathcal{D}}(x) \stackrel{\text{def}}{=} \overleftarrow{\mathcal{D}}(x) \stackrel{\text{def}}{=} (x, \lambda y.y) \quad \overrightarrow{\mathcal{D}}(\mathsf{op}(t))_1 \stackrel{\text{def}}{=} \mathsf{op}(\overrightarrow{\mathcal{D}}(t)_1) \quad \overleftarrow{\mathcal{D}}(\mathsf{op}(t))_1 \stackrel{\text{def}}{=} \mathsf{op}(\overleftarrow{\mathcal{D}}(t)_1)$$

$$\overrightarrow{\mathcal{D}}(\mathsf{op}(t))_2 \stackrel{\text{def}}{=} \lambda y.(D\mathsf{op})(\overrightarrow{\mathcal{D}}(t)_1)\,(\overrightarrow{\mathcal{D}}(t)_2\, y) \quad \overleftarrow{\mathcal{D}}(\mathsf{op}(t))_2 \stackrel{\text{def}}{=} \lambda y.\overleftarrow{\mathcal{D}}(t)_2\,((D\mathsf{op})^t(\overleftarrow{\mathcal{D}}(t)_1)\, y),$$

where we assume that we have chosen suitable terms $x : \mathbf{real}^n \vdash (D\mathsf{op})(x) : \mathbf{real}^n \to \mathbf{real}^m$ and $x : \mathbf{real}^n \vdash (D\mathsf{op})^t(x) : \mathbf{real}^m \to \mathbf{real}^n$ to represent the (multivariate) derivative and transposed (multivariate) derivative, respectively, of the primitive operation $\mathsf{op} : \mathbf{real}^n \to \mathbf{real}^m$.

For example, in case of multiplication $x : \mathbf{real}^n \vdash \mathsf{op}(x) = (*)(x) : \mathbf{real}$, we can choose $D(*)(x) = \lambda y : \mathbf{real}^2.\mathbf{swap}(x) \bullet y$ and $(D(*))^t(x) = \lambda y : \mathbf{real}.y \cdot \mathbf{swap}(x)$, where $\mathbf{swap}$ is a unary operation on $\mathbf{real}^2$ that swaps both components, $(\bullet)$ is a binary inner product operation on $\mathbf{real}^2$ and $(\cdot)$ is a binary scalar product operation for rescaling a vector in $\mathbf{real}^2$ by a real number .

To illustrate the difference between $\overrightarrow{\mathcal{D}}$ and $\overleftarrow{\mathcal{D}}$, consider the program $t = \mathsf{op}_2(\mathsf{op}_1(x))$ performing two operations in sequence. Then, $\overrightarrow{\mathcal{D}}(t)_1 = \mathsf{op}_2(\mathsf{op}_1(x)) = \overleftarrow{\mathcal{D}}(t)_1$ and (after $\beta$-reducing, for legibility)

$$\overrightarrow{\mathcal{D}}(t)_2 = \lambda y.(D\mathsf{op}_2)(\mathsf{op}_1(x))((D\mathsf{op}_1)(x)(y))$$

$$\overleftarrow{\mathcal{D}}(t)_2 = \lambda y.(D\mathsf{op}_1)^t(x)((D\mathsf{op}_2)^t(\mathsf{op}_1(x))(y)).$$

In general, $\overrightarrow{\mathcal{D}}$ computes the derivative of a program that is a composition of operations $\mathsf{op}_1, \ldots, \mathsf{op}_n$ as the composition $(D\mathsf{op}_1), \ldots, (D\mathsf{op}_n)$ of the (multivariate) derivatives, in the same order as the original computation. By constrast, $\overleftarrow{\mathcal{D}}$ computes the *transposed* derivative of such a composition of $\mathsf{op}_1, \ldots, \mathsf{op}_n$ as the composition of the transposed derivatives $(D\mathsf{op}_n)^t, \ldots, (D\mathsf{op}_1)^t$. Observe the *reversed order* compared to the original composition!

While this AD technique works on the limited first-order language we described, it is far from satisfying. Notably, it has the following two shortcomings:

1. it does not tell us how to perform AD on programs that involve tuples or operations of multiple arguments;
2. it does not tell us how to perform AD on higher-order programs, that is, programs involving $\lambda$-abstractions and applications.

The key contributions of this paper are its extension of this transformation (see §7) to apply to a full simply typed $\lambda$-calculus (of §3), and its proof that this transformation is correct (see §8).

Shortcoming 1 seems easy to address, at first sight. Indeed, as the (co)tangent vectors to a product of spaces are simply tuples of (co)tangent vectors, one would expect to define, for a product type $\tau * \sigma$,

$$\overrightarrow{\mathcal{D}}(\tau*\sigma) \stackrel{\text{def}}{=} (\overrightarrow{\mathcal{D}}(\tau)_1 * \overrightarrow{\mathcal{D}}(\sigma)_1, \overrightarrow{\mathcal{D}}(\tau)_2 * \overrightarrow{\mathcal{D}}(\sigma)_2) \qquad \overleftarrow{\mathcal{D}}(\tau*\sigma) \stackrel{\text{def}}{=} (\overleftarrow{\mathcal{D}}(\tau)_1 * \overleftarrow{\mathcal{D}}(\sigma)_1, \overleftarrow{\mathcal{D}}(\tau)_2 * \overleftarrow{\mathcal{D}}(\sigma)_2).$$

Indeed, this technique straightforwardly applies to forward mode AD:

$$\overrightarrow{\mathcal{D}}(\langle t, s\rangle) \stackrel{\text{def}}{=} (\langle \overrightarrow{\mathcal{D}}(t)_1, \overrightarrow{\mathcal{D}}(s)_1\rangle, \lambda y.\langle \overrightarrow{\mathcal{D}}(t)_2(y), \overrightarrow{\mathcal{D}}(s)_2(y)\rangle)$$

$$\overrightarrow{\mathcal{D}}(\mathbf{fst}\, t) \stackrel{\text{def}}{=} (\mathbf{fst}\, \overrightarrow{\mathcal{D}}(t)_1, \lambda y.\mathbf{fst}\, \overrightarrow{\mathcal{D}}(t)_2(y)) \qquad \overrightarrow{\mathcal{D}}(\mathbf{snd}\, t) \stackrel{\text{def}}{=} (\mathbf{snd}\, \overrightarrow{\mathcal{D}}(t)_1, \lambda y.\mathbf{snd}\, \overrightarrow{\mathcal{D}}(t)_2(y)).$$

For reverse mode AD, however, tuples already present challenges. Indeed, we would like to use the definitions below, but they require terms $\vdash \underline{0} : \tau$ and $t + s : \tau$ for any two $t, s : \tau$ for each type $\tau$:

$$\overleftarrow{\mathcal{D}}(\langle t, s\rangle) \stackrel{\text{def}}{=} (\langle \overleftarrow{\mathcal{D}}(t)_1, \overleftarrow{\mathcal{D}}(s)_1\rangle, \lambda y.\overleftarrow{\mathcal{D}}(t)_2\,(\mathbf{fst}\, y) + \overleftarrow{\mathcal{D}}(s)_2\,(\mathbf{snd}\, y))$$

$$\overleftarrow{\mathcal{D}}(\mathbf{fst}\, t) \stackrel{\text{def}}{=} (\mathbf{fst}\, \overleftarrow{\mathcal{D}}(t)_1, \lambda y.\langle \overleftarrow{\mathcal{D}}(t)_2(y), \underline{0}\rangle) \qquad \overleftarrow{\mathcal{D}}(\mathbf{snd}\, t) \stackrel{\text{def}}{=} (\mathbf{snd}\, \overleftarrow{\mathcal{D}}(t)_1, \lambda y.\langle \underline{0}, \overleftarrow{\mathcal{D}}(t)_2(y)\rangle).$$

These formulae capture the well-known issue of fanout translating to addition in reverse AD, caused by the contravariance of its second component [31]. Such $\underline{0}$ and $+$ could indeed be defined by induction on the structure of types, using $\underline{0}$ and $+$ at $\mathbf{real}^n$. However, more problematically, $\langle -, -\rangle$, $\mathbf{fst}\,-$ and $\mathbf{snd}\,-$ represent explicit uses of structural rules of contraction and weakening at types $\tau$, which, in a $\lambda$-calculus, can also be used *implicitly* in the typing context $\Gamma$. Thus, we should also make these implicit uses *explicit* to account for their presence in the code. Then, we can appropriately translate them into their "mirror image": we map the contraction-weakening comonoids to the monoid structures $(+, \underline{0})$.

**Insight 1.** *In functional define-then-run reverse AD, we need to make use of explicit structural rules and "mirror them", which we can do by first translating our language into combinators. This translation allows us to avoid the usual practice (e.g. [38]) of accumulating adjoints at run-time with mutable state: instead, we detect all adjoints to accumulate at compile-time.*

Put differently: we define AD on the syntactic category **Syn** with types $\tau$ as objects and $(\alpha)\beta\eta$-equivalence classes of programs $x : \tau \vdash t : \sigma$ as morphisms $\tau \to \sigma$.

Yet the question remains: why should this translation for tuples be correct? What is even less clear is how to address shortcoming 2. What should the spaces of tangents $\overrightarrow{\mathcal{D}}(\tau \to \sigma)_2$ and adjoints $\overleftarrow{\mathcal{D}}(\tau \to \sigma)_2$ look like? This is not something we are taught in Calculus 1.01. Instead, we again employ category theory:

**Insight 2.** *Follow where the categorical structure of the syntax leads you, as doing so produces principled definitions that are easy to prove correct.*

With the aim of categorical compositionality in mind, we note that our translations compose according to a sort of "syntactic chain-rule", which says that

$$\overrightarrow{\mathcal{D}}(t[{}^{s}/_{x}]) \stackrel{\text{def}}{=} (\overrightarrow{\mathcal{D}}(t)_1[{}^{\overrightarrow{\mathcal{D}}(s)_1}/_{x}], \lambda y.\overrightarrow{\mathcal{D}}(t)_2[{}^{\overrightarrow{\mathcal{D}}(s)_1}/_{x}](\overrightarrow{\mathcal{D}}(s)_2(y)))$$
$$\overleftarrow{\mathcal{D}}(t[{}^{s}/_{x}]) \stackrel{\text{def}}{=} (\overleftarrow{\mathcal{D}}(t)_1[{}^{\overleftarrow{\mathcal{D}}(s)_1}/_{x}], \lambda y.\overleftarrow{\mathcal{D}}(s)_2(\overleftarrow{\mathcal{D}}(t)_2(y)[{}^{\overleftarrow{\mathcal{D}}(s)_1}/_{x}])).$$

By the following trick, these equations are functoriality laws. Given a Cartesian closed category $(\mathcal{C}, \mathbb{1}, \times, \Rightarrow)$, define categories $\overrightarrow{\mathfrak{D}}[\mathcal{C}]$ and $\overleftarrow{\mathfrak{D}}[\mathcal{C}]$ as having objects pairs $(A_1, A_2)$ of objects $A_1, A_2$ of $\mathcal{C}$ and morphisms

$$\overrightarrow{\mathfrak{D}}[\mathcal{C}]((A_1, A_2), (B_1, B_2)) \stackrel{\text{def}}{=} \mathcal{C}(A_1, B_1) \times \mathcal{C}(A_1, A_2 \Rightarrow B_2)$$
$$\overleftarrow{\mathfrak{D}}[\mathcal{C}]((A_1, A_2), (B_1, B_2)) \stackrel{\text{def}}{=} \mathcal{C}(A_1, B_1) \times \mathcal{C}(A_1, B_2 \Rightarrow A_2).$$

Both have identities $\mathrm{id}_{(A_1,A_2)} \stackrel{\text{def}}{=} (\mathrm{id}_{A_1}, \Lambda(\pi_2))$, where we write $\Lambda$ for categorical currying and $\pi_2$ for the second projection. Composition in $\overrightarrow{\mathfrak{D}}[\mathcal{C}]$ and $\overleftarrow{\mathfrak{D}}[\mathcal{C}]$, respectively, of $(A_1, A_2) \xrightarrow{(k_1,k_2)} (B_1, B_2) \xrightarrow{(l_1,l_2)} (C_1, C_2)$ are

$$(k_1, k_2); (l_1, l_2) \stackrel{\text{def}}{=} (k_1; l_1, \lambda a_1 : A_1.\lambda a_2 : A_2.l_2(k_1(a_1))(k_2(a_1, a_2)))$$
$$(k_1, k_2); (l_1, l_2) \stackrel{\text{def}}{=} (k_1; l_1, \lambda a_1 : A_1.\lambda c_2 : C_2.k_2(a_1)(l_2(k_1(a_1), c_2))),$$

where we work in the internal language of $\mathcal{C}$. Then, we have defined two functors:

$$\overrightarrow{\mathcal{D}} : \mathbf{Syn}_1 \to \overrightarrow{\mathfrak{D}}[\mathbf{Syn}] \qquad\qquad \overleftarrow{\mathcal{D}} : \mathbf{Syn}_1 \to \overleftarrow{\mathfrak{D}}[\mathbf{Syn}],$$

where we write $\mathbf{Syn}_1$ for the syntactic category of our restrictive first-order language, and we write **Syn** for that of the full $\lambda$-calculus. We would like to extend these to functors

$$\mathbf{Syn} \to \overrightarrow{\mathfrak{D}}[\mathbf{Syn}] \qquad\qquad \mathbf{Syn} \to \overleftarrow{\mathfrak{D}}[\mathbf{Syn}].$$

$\overrightarrow{\mathfrak{D}}[\mathcal{C}]$ turns out to be a category with finite products, given by $(A_1, A_2) \times (B_1, B_2) = (A_1 \times B_1, A_2 \times B_2)$. Thus, we can easily extend $\overrightarrow{\mathcal{D}}$ to apply to an extension of $\mathbf{Syn}_1$ with tuples by extending the functor in the unique structure-preserving way. However, $\overleftarrow{\mathfrak{D}}[\mathbf{Syn}]$ does not have products and neither $\overrightarrow{\mathfrak{D}}[\mathbf{Syn}]$ nor $\overleftarrow{\mathfrak{D}}[\mathbf{Syn}]$ supports function types. (The reason turns out to be that not all functions are linear in the sense of respecting $\underline{0}$ and $+$.) Therefore, the categorical structure does not give us guidance on how to extend our translation to all of **Syn**.

**Insight 3.** *Linear types can help. By using a more fine-grained type system, we can capture the linearity of the derivative. As a result, we can phrase AD on our full language simply as the unique structure-preserving functor that extends the uncontroversial definitions given so far.*

To implement this insight, we extend our $\lambda$-calculus to a language **LSyn** with limited linear types (in §4): linear function types $\multimap$ and a kind of multiplicative conjunction $!(-) \otimes (-)$, in the sense of the enriched effect calculus [14]. The algebraic effect giving rise to these linear types, in this instance, is that of the theory of commutative monoids. As we have seen, such monoids are intimately related to reverse AD. Consequently, we demand that every $f$ with a linear function type $\tau \multimap \sigma$ is indeed linear, in the sense that $f\,\underline{0} = \underline{0}$ and $f\,(t+s) = (f\,t) + (f\,s)$. For the categorically inclined reader: that is, we enrich **LSyn** over the category of commutative monoids.

Now, we can give more precise types to our derivatives, as we know they are linear functions: for $x : \tau \vdash t : \sigma$, we have $x : \overrightarrow{\mathcal{D}}(\tau)_1 \vdash \overrightarrow{\mathcal{D}}(t)_2 : \overrightarrow{\mathcal{D}}(\tau)_2 \multimap \overrightarrow{\mathcal{D}}(\sigma)_2$ and $x : \overleftarrow{\mathcal{D}}(\tau)_1 \vdash \overleftarrow{\mathcal{D}}(t)_2 : \overleftarrow{\mathcal{D}}(\sigma)_2 \multimap \overleftarrow{\mathcal{D}}(\tau)_2$. Therefore, given any model $\mathcal{L}$ of our linear type theory, we generalise our previous construction of the categories $\overrightarrow{\mathfrak{D}}[\mathcal{L}]$ and $\overleftarrow{\mathfrak{D}}[\mathcal{L}]$, but now we work with linear functions in the second component. Unlike before, both $\overrightarrow{\mathfrak{D}}[\mathcal{L}]$ and $\overleftarrow{\mathfrak{D}}[\mathcal{L}]$ are now Cartesian closed (by §6)!

Thus, we find the following corollary, by the universal property of **Syn**. This property states that any well-typed choice of interpretations $F(\mathsf{op})$ of the primitive operations in a Cartesian closed category $\mathcal{C}$ extends to a unique Cartesian closed functor $F : \mathbf{Syn} \to \mathcal{C}$. It gives a principled definition of AD and explains in what sense reverse AD is the "mirror image" of forward AD.

**Corollary** (Definition of AD, §7)**.** *Once we fix the interpretation of the primitives operations* $\mathsf{op}$ *to their respective derivatives and transposed derivatives, we obtain unique structure-preserving forward and reverse AD functors* $\overrightarrow{\mathcal{D}} : \mathbf{Syn} \to \overrightarrow{\mathfrak{D}}[\mathbf{LSyn}]$ *and* $\overleftarrow{\mathcal{D}} : \mathbf{Syn} \to \overleftarrow{\mathfrak{D}}[\mathbf{LSyn}]$.

In particular, the following definitions are forced on us by the theory:

**Insight 4.** *For reverse AD, an adjoint at function type* $\tau \to \sigma$*, needs to keep track of the incoming adjoints* $v$ *of type* $\overleftarrow{\mathcal{D}}(\sigma)_2$ *for each a primal* $x$ *of type* $\overleftarrow{\mathcal{D}}(\tau)_1$ *on which we call the function. We store these pairs* $(x, v)$ *in the type* $!\overleftarrow{\mathcal{D}}(\tau)_1 \otimes \overleftarrow{\mathcal{D}}(\sigma)_2$ *(which we will see is essentially a quotient of a list of pairs of type* $\overleftarrow{\mathcal{D}}(\tau)_1 * \overleftarrow{\mathcal{D}}(\sigma)_2$*). Less surprisingly, for forward AD, a tangent at function type* $\tau \to \sigma$ *consists of a function sending each argument primal of type* $\overrightarrow{\mathcal{D}}(\tau)_1$ *to the outgoing tangent of type* $\overrightarrow{\mathcal{D}}(\sigma)_2$*.*

$$\overrightarrow{\mathcal{D}}(\tau \to \sigma) \stackrel{\text{def}}{=} (\overrightarrow{\mathcal{D}}(\tau)_1 \to (\overrightarrow{\mathcal{D}}(\sigma)_1 * (\overrightarrow{\mathcal{D}}(\tau)_2 \multimap \overrightarrow{\mathcal{D}}(\sigma)_2)), \overrightarrow{\mathcal{D}}(\tau)_1 \to \overrightarrow{\mathcal{D}}(\sigma)_2)$$
$$\overleftarrow{\mathcal{D}}(\tau \to \sigma) \stackrel{\text{def}}{=} (\overleftarrow{\mathcal{D}}(\tau)_1 \to (\overleftarrow{\mathcal{D}}(\sigma)_1 * (\overleftarrow{\mathcal{D}}(\sigma)_2 \multimap \overleftarrow{\mathcal{D}}(\tau)_2)), !\overleftarrow{\mathcal{D}}(\tau)_1 \otimes \overleftarrow{\mathcal{D}}(\sigma)_2)$$

With these definitions in place, we turn to the correctness of the source-code transformations. To phrase correctness, we first need to construct a suitable denotational semantics with an uncontroversial notion of semantic differentiation. A technical challenge arises, as the usual calculus setting of Euclidean spaces (or manifolds) and smooth functions cannot interpret higher-order functions. To solve this problem, we work with a conservative extension of this standard calculus setting (see §5): the category **Diff** of diffeological spaces. We model our types as diffeological spaces, and programs as smooth functions. By keeping track of a commutative monoid structure on these spaces, we are also able to interpret the required linear types. We write $\mathbf{Diff}_{\mathbf{CM}}$ for this "linear" category of commutative diffeological monoids and smooth monoid homomorphisms.

By the universal properties of the syntax, we obtain canonical, structure-preserving functors $\llbracket - \rrbracket : \mathbf{LSyn} \to \mathbf{Diff}_{\mathbf{CM}}$ and $\llbracket - \rrbracket : \mathbf{Syn} \to \mathbf{Diff}$ once we fix interpretations $\mathbb{R}^n$ of $\mathbf{real}^n$ and well-typed interpretations $\llbracket \mathsf{op} \rrbracket$ for each operation $\mathsf{op}$. These functors define a semantics for our language.

Having constructed the semantics, we can turn to the correctness proof (of §8). Because calculus does not provide an unambiguous notion of derivative at function spaces, we cannot prove that the AD transformations correctly implement mathematical derivatives by plain induction on the syntax. Instead, we use a logical relations argument over the semantics, which we phrase categorically:

**Insight 5.** *Once we show that the derivatives of primitive operations* $\mathsf{op}$ *are correctly implemented, correctness of derivatives of other programs follows from a standard logical relations construction over the semantics that relates a curve to its (co)tangent curve. By the chain-rule, all programs respect the logical relations.*

To show correctness of forward AD, we construct a category $\overrightarrow{\mathbf{SScone}}$ whose objects are triples $((X,(Y_1,Y_2)),P)$ of an object $X$ of $\mathbf{Diff}$, an object $(Y_1,Y_2)$ of $\overrightarrow{\mathfrak{D}}[\mathbf{Diff}_{\mathbf{CM}}]$ and a predicate $P$ on $\mathbf{Diff}(\mathbb{R},X) \times \overrightarrow{\mathfrak{D}}[\mathbf{Diff}_{\mathbf{CM}}]((\mathbb{R},\mathbb{R}),(Y_1,Y_2))$. It has morphisms $((X,(Y_1,Y_2)),P) \xrightarrow{(f,(g,h))} ((X',(Y_1',Y_2')),P')$, which are a pair of morphisms $X \xrightarrow{f} X'$ and $(Y_1,Y_2) \xrightarrow{(g,h)} (Y_1',Y_2')$ such that for any $(\gamma,(\delta_1,\delta_2)) \in P$, we have that $(\gamma;f,(\delta_1,\delta_2);(g,h)) \in P'$. $\overrightarrow{\mathbf{SScone}}$ is a standard category of logical relations, or subscone, and it is widely known to inherit the Cartesian closure of $\mathbf{Diff} \times \overrightarrow{\mathfrak{D}}[\mathbf{Diff}_{\mathbf{CM}}]$ (see §§8.1). It also comes equipped with a Cartesian closed functor $\overrightarrow{\mathbf{SScone}} \to \mathbf{Diff} \times \overrightarrow{\mathfrak{D}}[\mathbf{Diff}_{\mathbf{CM}}]$. Therefore, once we fix predicates $P^f_{\mathbf{real}^n}$ on $(\llbracket - \rrbracket, \overrightarrow{\mathfrak{D}}[\llbracket - \rrbracket])(\mathbf{real}^n)$ and show that all operations $\mathsf{op}$ respect these predicates, it follows that our denotational semantics lifts to give a unique structure-preserving functor $\mathbf{Syn} \xrightarrow{(\!|-|\!)^f} \overrightarrow{\mathbf{SScone}}$, such that the left diagram below commutes (by the universal property of $\mathbf{Syn}$).

$$
\begin{array}{ccc}
\mathbf{Syn} & \xrightarrow{(\mathrm{id},\overrightarrow{\mathcal{D}})} & \mathbf{Syn} \times \overrightarrow{\mathfrak{D}}[\mathbf{LSyn}] \\
{\scriptstyle (\!|-|\!)^f}\downarrow & & \downarrow{\scriptstyle \llbracket - \rrbracket \times \overrightarrow{\mathfrak{D}}[\llbracket - \rrbracket]} \\
\overrightarrow{\mathbf{SScone}} & \longrightarrow & \mathbf{Diff} \times \overrightarrow{\mathfrak{D}}[\mathbf{Diff}_{\mathbf{CM}}]
\end{array}
\qquad
\begin{array}{ccc}
\mathbf{Syn} & \xrightarrow{(\mathrm{id},\overleftarrow{\mathcal{D}})} & \mathbf{Syn} \times \overleftarrow{\mathfrak{D}}[\mathbf{LSyn}] \\
{\scriptstyle (\!|-|\!)^r}\downarrow & & \downarrow{\scriptstyle \llbracket - \rrbracket \times \overleftarrow{\mathfrak{D}}[\llbracket - \rrbracket]} \\
\overleftarrow{\mathbf{SScone}} & \longrightarrow & \mathbf{Diff} \times \overleftarrow{\mathfrak{D}}[\mathbf{Diff}_{\mathbf{CM}}]
\end{array}
$$

Consequently, we can work with $P^f_{\mathbf{real}^n} \stackrel{\mathrm{def}}{=} \{(f,(g,h)) \mid g = f \text{ and } h = Df\}$, where we write $Df(x)(v)$ for the multivariate calculus derivative of $f$ at a point $x$ evaluated at a tangent vector $v$. By an application of the chain rule for differentiation, we see that every $\mathsf{op}$ respects this predicate, as long as $\llbracket D\mathsf{op} \rrbracket = D\llbracket \mathsf{op} \rrbracket$. The commuting of our diagram then virtually establishes the correctness of forward AD. The only remaining step in the argument is to note that any tangent vector at $\llbracket \tau \rrbracket \cong \mathbb{R}^N$, for first-order $\tau$, can be represented by a curve $\mathbb{R} \to \llbracket \tau \rrbracket$. For reverse AD, the same construction works, if $\llbracket D\mathsf{op}^t \rrbracket = D\llbracket \mathsf{op} \rrbracket^t$, by replacing $\overrightarrow{\mathfrak{D}}[-]$ with $\overleftarrow{\mathfrak{D}}[-]$ and $\overrightarrow{\mathcal{D}}$ with $\overleftarrow{\mathcal{D}}$. We can then choose $P^r_{\mathbf{real}^n} \stackrel{\mathrm{def}}{=} \left\{(f,(g,h)) \mid g = f \text{ and } h = x \mapsto (Df(x))^t\right\}$, as the predicates for constructing $(\!|\mathbf{real}^n|\!)^r$, where we write $A^t$ for the matrix transpose of $A$. We obtain our main theorem, which crucially holds even for $t$ that involve higher-order subprograms.

**Theorem** (Correctness of AD, Thm. 1)**.** *For any typed term $x : \tau \vdash t : \sigma$ in* **Syn** *between first-order types $\tau, \sigma$, we have that*

$$[\![\overrightarrow{\mathcal{D}}(t)_2]\!](x) = D[\![t]\!](x) \quad \textit{and} \quad [\![\overleftarrow{\mathcal{D}}(t)_2]\!](x) = D[\![t]\!](x)^t.$$

Next, we address the practicality of our method (in §9). The code transformations we employ are not too daunting to implement. It is well-known how to mechanically translate $\lambda$-calculus and functional languages into a (categorical) combinatory form [12]. However, the implementation of the required linear types presents a challenge. Indeed, types like $!(-) \otimes (-)$ and $(-) \multimap (-)$ are absent from languages such as Haskell and O'Caml. Luckily, in this instance, we can implement them using abstract data types by using a (basic) module system:

**Insight 6.** *Under the hood, $!\tau \otimes \sigma$ can consist of a list of values of type $\tau{*}\sigma$. Its API ensures that the list order and the difference between $xs +\!\!+ [(t, s), (t, s')]$ $+\!\!+\, ys$ and $xs +\!\!+ [(t, s + s')] +\!\!+ ys$ cannot be observed: as such, it is a quotient type. Meanwhile, $\tau \multimap \sigma$ can be implemented as a standard function type $\tau \to \sigma$ with a limited API that enforces that we can only ever construct linear functions: as such, it is a subtype.*

We phrase the correctness proof of the AD transformations in elementary terms, such that it holds in the applied setting where we use abstract types to implement linear types. We show that our correctness results are meaningful, as they make use of a denotational semantics that is adequate with respect to the standard operational semantics. Finally, to stress the applicability of our method, we show that it extends to higher-order (primitive) operations, such as **map**.

### 3 $\boldsymbol{\lambda}$-Calculus as a Source Language for AD

As a source language for our AD translations, we can begin with a standard, simply typed $\lambda$-calculus which has ground types $\mathbf{real}^n$ of statically sized arrays of $n$ real numbers, for all $n \in \mathbb{N}$, and sets $\mathsf{Op}^m_{n_1,\ldots,n_k}$ of primitive operations $\mathsf{op}$ for all $k, m, n_1, \ldots, n_k \in \mathbb{N}$. These operations will be interpreted as smooth functions $(\mathbb{R}^{n_1} \times \ldots \times \mathbb{R}^{n_k}) \to \mathbb{R}^m$. Examples to keep in mind for $\mathsf{op}$ include

- constants $\underline{c} \in \mathsf{Op}^n$ for each $c \in \mathbb{R}^n$, for which we slightly abuse notation and write $\underline{c}(\langle\rangle)$ as $\underline{c}$;
- elementwise addition and product $(+), (*) \in \mathsf{Op}^n_{n,n}$ and matrix-vector product $(\star) \in \mathsf{Op}^n_{n \cdot m, m}$;
- operations for summing all the elements in an array: $\mathrm{sum} \in \mathsf{Op}^1_n$;
- some non-linear functions like the sigmoid function $\varsigma \in \mathsf{Op}^1_1$.

We intentionally present operations in a schematic way, as primitive operations tend to form a collection that is added to in a by-need fashion, as an AD library develops. The precise operations needed will depend on the applications, but, in statistics and machine learning applications, $\mathsf{Op}$ tends to include a mix of multi-dimensional linear algebra operations and mostly one-dimensional non-linear functions. A typical library for use in machine learning would work with multi-dimensional arrays (sometimes called "tensors"). We focus here on one-dimensional arrays as the issues of how precisely to represent the arrays are orthogonal to the concerns of our development.

The types $\tau, \sigma, \rho$ and terms $t, s, r$ of our AD source language are as follows:

$$
\begin{array}{llllll}
\tau,\sigma,\rho & ::= & & \text{types} & \mid\ \tau_1 {*} \tau_2 & \text{binary product} \\
 & \mid & \mathbf{real}^n & \text{real arrays} & \mid\ \tau \to \sigma & \text{function} \\
 & \mid & \mathbf{1} & \text{nullary product} & & \\
t,s,r & ::= & & \text{terms} & \mid\ \mathbf{fst}\, t \ \mid \mathbf{snd}\, t & \text{product projections} \\
 & & x & \text{variable} & \mid\ \lambda x.t & \text{function abstraction} \\
 & \mid & \mathsf{op}(t) & \text{operations} & \mid\ t\, s & \text{function application} \\
 & \mid & \langle\rangle \ \mid \langle t, s\rangle & \text{product tuples} & &
\end{array}
$$

The typing rules are in Fig. 1, where we write $\mathbf{Dom}(\mathsf{op}) \stackrel{\text{def}}{=} \mathbf{real}^{n_1} {*} \ldots {*} \mathbf{real}^{n_k}$ for an operation $\mathsf{op} \in \mathsf{Op}^m_{n_1,\ldots,n_k}$. We employ the usual syntactic sugar $\mathbf{let}\, x = t\, \mathbf{in}\, s \stackrel{\text{def}}{=} (\lambda x.s)\, t$ and write $\mathbf{real}$ for $\mathbf{real}^1$. As Fig. 2 displays, we consider the terms of our language up to the standard $\beta\eta$-theory. We could consider further equations for our operations, but we do not as we will not need them.

This standard $\lambda$-calculus is widely known to be equivalent to the free Cartesian closed category **Syn** generated by the objects $\mathbf{real}^n$ and the morphisms $\mathsf{op}$. **Syn** effectively represents programs as (categorical) combinators, also known as "point-free style" in the functional programming community. Indeed, there are well-studied mechanical translations from the $\lambda$-calculus to the free Cartesian closed category (and back) [26,13]. The translation from **Syn** to $\lambda$-calculus is self-evident, while the translation in the opposite direction is straightforward after we first convert our $\lambda$-terms to de Bruijn indexed form. Concretely,

– **Syn** has types $\tau, \sigma, \rho$ objects;
– **Syn** has morphisms $t \in \mathbf{Syn}(\tau, \sigma)$ which are in 1-1 correspondence with terms $x : \tau \vdash t : \sigma$ up to $\beta\eta$-equivalence (which includes $\alpha$-equivalence); explicitly, they can be represented by
  - identities: $\mathrm{id}_\tau \in \mathbf{Syn}(\tau, \tau)$ (cf., variables up to $\alpha$-equivalence);
  - composition: $t; s \in \mathbf{Syn}(\tau, \rho)$ for any $t \in \mathbf{Syn}(\tau, \sigma)$ and $s \in \mathbf{Syn}(\sigma, \rho)$ (corresponding to the capture avoiding substitution $s[{}^t/_y]$ if we represent $x : \tau \vdash t : \sigma$ and $y : \sigma \vdash s : \rho$);
  - terminal morphisms: $\langle\rangle_\tau \in \mathbf{Syn}(\tau, \mathbf{1})$;
  - product pairing: $\langle t, s\rangle \in \mathbf{Syn}(\tau, \sigma {*} \rho)$ for any $t \in \mathbf{Syn}(\tau, \sigma)$ and $s \in \mathbf{Syn}(\tau, \rho)$;
  - product projections: $\mathbf{fst}_{\tau,\sigma} \in \mathbf{Syn}(\tau {*} \sigma, \tau)$ and $\mathbf{snd}_{\tau,\sigma} \in \mathbf{Syn}(\tau {*} \sigma, \sigma)$;

$$
\frac{((x:\tau)\in\Gamma)}{\Gamma\vdash x:\tau}
\qquad
\frac{\Gamma\vdash t:\mathbf{Dom}(\mathsf{op})\quad (\mathsf{op}\in\mathsf{Op}^m_{n_1,\ldots,n_k})}{\Gamma\vdash \mathsf{op}(t):\mathbf{real}^m}
\qquad
\frac{}{\Gamma\vdash\langle\rangle:\mathbf{1}}
\qquad
\frac{\Gamma\vdash t:\tau\quad \Gamma\vdash s:\sigma}{\Gamma\vdash\langle t,s\rangle:\tau{*}\sigma}
$$

$$
\frac{\Gamma\vdash t:\tau{*}\sigma}{\Gamma\vdash \mathbf{fst}\,t:\tau}
\qquad
\frac{\Gamma\vdash t:\tau{*}\sigma}{\Gamma\vdash \mathbf{snd}\,t:\sigma}
\qquad
\frac{\Gamma,x:\tau\vdash t:\sigma}{\Gamma\vdash\lambda x.t:\tau\to\sigma}
\qquad
\frac{\Gamma\vdash t:\sigma\to\tau\qquad \Gamma\vdash s:\sigma}{\Gamma\vdash t\,s:\tau}
$$

**Fig. 1.** Typing rules for the AD source language.

$$t = \langle\rangle \qquad \mathbf{fst}\,\langle t, s\rangle = t \quad \mathbf{snd}\,\langle t, s\rangle = s \quad t = \langle \mathbf{fst}\,t, \mathbf{snd}\,t\rangle \quad (\lambda x.t)\,s = t[{}^{s}/_{x}] \quad t \stackrel{\#x}{=} \lambda x.t\,x$$

**Fig. 2.** Standard $\beta\eta$-laws for products and functions. We write $\stackrel{\#x_1,\ldots,x_n}{=}$ to indicate that the variables $x_1, \ldots, x_n$ need to be fresh in the left hand side. Equations hold on pairs of terms of the same type. As usual, we only distinguish terms up to $\alpha$-renaming of bound variables.

- function evaluation: $\mathrm{ev}_{\tau,\sigma} \in \mathbf{Syn}((\tau \to \sigma)*\tau, \sigma)$;
- currying: $\Lambda_{\tau,\sigma,\rho}(t) \in \mathbf{Syn}(\tau, \sigma \to \rho)$ for any $t \in \mathbf{Syn}(\tau*\sigma, \rho)$;
- operations: $\mathsf{op} \in \mathbf{Syn}(\mathbf{real}^{n_1}*..*\mathbf{real}^{n_k}, \mathbf{real}^m)$ for any $\mathsf{op} \in \mathsf{Op}^m_{n_1,..,n_k}$.

– all subject to the usual equations of a Cartesian closed category [26].

$\mathbf{1}$ and $*$ give finite products in **Syn**, while $\to$ gives categorical exponentials.

**Syn** has the following universal property: for any Cartesian closed category $(\mathcal{C}, \mathbb{1}, \times, \Rightarrow)$, we obtain a unique Cartesian closed functor $F : \mathbf{Syn} \to \mathcal{C}$, once we choose objects $F\mathbf{real}^n$ of $\mathcal{C}$ as well as, for each $\mathsf{op} \in \mathsf{Op}^m_{n_1,\ldots,n_k}$, make well-typed choices of $\mathcal{C}$-morphisms $F\mathsf{op} : (F\mathbf{real}^{n_1} \times \ldots \times F\mathbf{real}^{n_k}) \to F\mathbf{real}^m$.

### 4 Linear λ-Calculus as an Idealised AD Target Language

As a target language for our AD source code transformations, we consider a language that extends the language of §3 with limited linear types. We could opt to work with a full linear logic as in [6] or [4]. Instead, however, we will only include the bare minimum of linear type formers that we actually need to phrase the AD transformations. The resulting language is closely related to, but more minimal than, the Enriched Effect Calculus of [14]. We limit our language in this way because we want to stress that the resulting code transformations can easily be implemented in existing functional languages such as Haskell or O'Caml. As we discuss in §9, the idea will be to make use of a module system to implement the required linear types as abstract data types.

In our idealised target language, we consider *linear types* (aka computation types) $\underline{\tau}$, $\underline{\sigma}$, $\underline{\rho}$, in addition to the *Cartesian types* (aka value types) $\tau$, $\sigma$, $\rho$ that we have considered so far. We think of Cartesian types as denoting spaces and linear types as denoting spaces equipped with an algebraic structure. As we are interested in studying differentiation, the relevant space structure in this instance is a geometric structure that suffices to define differentiability. Meanwhile, the relevant algebraic structure on linear types turns out to be that of a commutative monoid, as this algebraic structure is needed to phrase automatic differentiation algorithms. Indeed, we will use the linear types to denote spaces of (co)tangent vectors to the spaces of primals denoted by Cartesian types. These spaces of (co)tangents form a commutative monoid under addition.

Concretely, we extend the types and terms of our language as follows:

| | | | | | |
|---|---|---|---|---|---|
| $\underline{\tau}, \underline{\sigma}, \underline{\rho}$ | $::=$ | linear types | $\mid$ | $\underline{\tau}*\underline{\sigma}$ | binary product |
| | $\mid$ $\underline{\mathbf{real}}^n$ | real array | $\mid$ | $\tau \to \underline{\sigma}$ | function |
| | $\mid$ $\underline{\mathbf{1}}$ | unit type | $\mid$ | $!\tau \otimes \underline{\sigma}$ | tensor product |
| $\tau, \sigma, \rho$ | $::=$ | Cartesian types | $\mid$ | $\underline{\tau} \multimap \underline{\sigma}$ | linear function |
| | $\mid$ $\ldots$ | as in §3 | | | |

$$\frac{}{\Gamma; x:\underline{\tau} \vdash x:\underline{\tau}} \quad \frac{\Gamma \vdash t:\mathbf{Dom}(\mathsf{lop}) \quad \Gamma; x:\underline{\tau} \vdash s:\mathbf{LDom}(\mathsf{lop}) \quad (\mathsf{lop} \in \mathsf{LOp}^m_{n_1,\ldots,n_k;n'_1,\ldots,n'_l})}{\Gamma; x:\underline{\tau} \vdash \mathsf{lop}(t;s):\underline{\mathbf{real}}^m}$$

$$\frac{}{\Gamma; x:\underline{\tau} \vdash \langle\rangle:\underline{\mathbf{1}}} \quad \frac{\Gamma; x:\underline{\tau} \vdash t:\underline{\sigma} \quad \Gamma; x:\underline{\tau} \vdash s:\underline{\rho}}{\Gamma; x:\underline{\tau} \vdash \langle t,s\rangle:\underline{\sigma}{*}\underline{\rho}} \quad \frac{\Gamma; x:\underline{\tau} \vdash t:\underline{\sigma}{*}\underline{\rho}}{\Gamma; x:\underline{\tau} \vdash \mathbf{fst}\, t:\underline{\sigma}} \quad \frac{\Gamma; x:\underline{\tau} \vdash t:\underline{\sigma}{*}\underline{\rho}}{\Gamma; x:\underline{\tau} \vdash \mathbf{snd}\, t:\underline{\rho}}$$

$$\frac{\Gamma, y:\sigma; x:\underline{\tau} \vdash t:\underline{\rho}}{\Gamma; x:\underline{\tau} \vdash \lambda y.t:\sigma \to \underline{\rho}} \quad \frac{\Gamma; x:\underline{\tau} \vdash t:\sigma \to \underline{\rho} \quad \Gamma \vdash s:\sigma}{\Gamma; x:\underline{\tau} \vdash t\, s:\underline{\rho}} \quad \frac{\Gamma \vdash t:\sigma \quad \Gamma; x:\underline{\tau} \vdash s:\underline{\rho}}{\Gamma; x:\underline{\tau} \vdash !t \otimes s : !\sigma \otimes \underline{\rho}}$$

$$\frac{\Gamma; x:\underline{\tau} \vdash t : !\sigma \otimes \underline{\rho} \quad \Gamma, y:\sigma; z:\underline{\rho} \vdash s:\underline{\rho}'}{\Gamma; x:\underline{\tau} \vdash \mathbf{case}\, t\, \mathbf{of}\, !y \otimes z \to s:\underline{\rho}'} \quad \frac{\Gamma; x:\underline{\tau} \vdash t:\underline{\sigma}}{\Gamma \vdash \underline{\lambda} x.t:\underline{\tau} \multimap \underline{\sigma}}$$

$$\frac{\Gamma \vdash t:\underline{\rho} \multimap \underline{\sigma} \quad \Gamma; x:\underline{\tau} \vdash s:\underline{\rho}}{\Gamma; x:\underline{\tau} \vdash t\{s\}:\underline{\sigma}} \quad \frac{}{\Gamma; x:\underline{\tau} \vdash \underline{0}:\underline{\sigma}} \quad \frac{\Gamma; x:\underline{\tau} \vdash t:\underline{\sigma} \quad \Gamma; x:\underline{\tau} \vdash s:\underline{\sigma}}{\Gamma; x:\underline{\tau} \vdash t+s:\underline{\sigma}}$$

**Fig. 3.** Typing rules for the idealised AD target language with linear types.

| | | | | |
|---|---|---|---|---|
| $t, s, r ::=$ terms | | $\mid\ !t \otimes s \mid \mathbf{case}\, t\, \mathbf{of}\, !y \otimes z \to s$ | tensor product |
| $\mid \ \ldots$ | as in §3 | $\mid\ \underline{\lambda} x.t \mid t\{s\}$ | abstraction/appl. |
| $\mid\ \mathsf{lop}(t;s)$ | linear op. | $\mid\ \underline{0} \mid t+s$ | monoid structure. |

We work with linear operations $\mathsf{lop} \in \mathsf{LOp}^m_{n_1,\ldots,n_k;n'_1,\ldots,n'_l}$, which are intended to represent functions which are linear (in the sense of respecting $\underline{0}$ and $+$) in the last $l$ arguments but not in the first $k$. We write $\mathbf{Dom}(\mathsf{lop}) \stackrel{\text{def}}{=} \mathbf{real}^{n_1} * \ldots * \mathbf{real}^{n_k}$ and $\mathbf{LDom}(\mathsf{lop}) \stackrel{\text{def}}{=} \underline{\mathbf{real}}^{n'_1} * \ldots * \underline{\mathbf{real}}^{n'_l}$ for $\mathsf{lop} \in \mathsf{LOp}^m_{n_1,\ldots,n_k;n'_1,\ldots,n'_l}$. These operations can include e.g. dense and sparse matrix-vector multiplications. Their purpose is to serve as primitives to implement derivatives $D\mathsf{op}(x;y)$ and $(D\mathsf{op})^t(x;y)$ of the operations $\mathsf{op}$ from the source language as terms that are linear in $y$.

In addition to the judgement $\Gamma \vdash t : \tau$, which we encountered in §3, we now consider an additional judgement $\Gamma; x : \underline{\tau} \vdash t : \underline{\sigma}$. While we think of the former as denoting a (structure-preserving) function between spaces, we think of the latter as a (structure-preserving) function from the space which $\Gamma$ denotes to the space of (structure-preserving) monoid homomorphisms from the denotation of $\underline{\tau}$ to that of $\underline{\sigma}$. In this instance, "structure-preserving" will mean differentiable.

Fig. 3 displays the typing rules of our language. We consider the terms of this language up to the $\beta\eta+$-equational theory of Fig. 4. It includes $\beta\eta$-rules as well as commutative monoid and homomorphism laws.

| | |
|---|---|
| $\mathbf{case}\, !t \otimes s\, \mathbf{of}\, !x \otimes y \to r = r[{}^t/_x, {}^s/_y]$ | $t[{}^s/_x] \stackrel{\#y,z}{=} \mathbf{case}\, s\, \mathbf{of}\, !y \otimes z \to t[{}^{!y \otimes z}/_x]$ |
| $(\underline{\lambda} x.t)\{s\} = t[{}^s/_x]$ | $t \stackrel{\#x}{=} \underline{\lambda} x.t\{x\}$ |
| $t + \underline{0} = t \qquad \underline{0} + t = t$ | $(t+s)+r = t+(s+r) \qquad t+s = s+t$ |
| $(\Gamma; x:\underline{\tau} \vdash t:\underline{\sigma}) \Rightarrow t[{}^{\underline{0}}/_x] = \underline{0}$ | $(\Gamma; x:\underline{\tau} \vdash t:\underline{\sigma}) \Rightarrow t[{}^{s+r}/_x] = t[{}^s/_x] + t[{}^r/_x]$ |

**Fig. 4.** Equational rules for the idealised, linear AD language, which we use on top of the rules of Fig. 2. In addition to standard $\beta\eta$-rules for $!(-) \otimes (-)$- and $\multimap$-types, we add rules making $(\underline{0}, +)$ into a commutative monoid on the terms of each linear type as well as rules which say that terms of linear types are homomorphisms in their linear variable. Equations hold on pairs of terms of the same type.

## 5 Semantics of the Source and Target Languages

### 5.1 Preliminaries

**Category theory** We assume familiarity with categories, functors, natural transformations, and their theory of (co)limits and adjunctions. We write:

- unary, binary, and $I$-ary products as $\mathbb{1}$, $X_1 \times X_2$, and $\prod_{i\in I} X_i$, writing $\pi_i$ for the projections and $()$, $(x_1, x_2)$, and $(x_i)_{i\in I}$ for the tupling maps;
- unary, binary, and $I$-ary coproducts as $\mathbb{0}$, $X_1 + X_2$, and $\sum_{i\in I} X_i$, writing $\iota_i$ for the injections and $[]$, $[x_1, x_2]$, and $[x_i]_{i\in I}$ for the cotupling maps;
- exponentials as $Y \Rightarrow X$, writing $\Lambda$ and $\mathrm{ev}$ for currying and evaluation.

**Monoids** We assume familiarity with the category **CMon** of commutative monoids $X = (|X|, 0_X, +_X)$, such as $\underline{\mathbb{R}}^n \stackrel{\mathrm{def}}{=} (\mathbb{R}^n, 0, +)$, their cartesian product $X \times Y$, tensor product $X \otimes Y$, and the free monoid $!S$ on a set $S$ (write $\delta$ for the inclusion $S \hookrightarrow |!S|$). We will sometimes write $\sum_{i=1}^n x_i$ for $((x_1+x_2)+\ldots)\ldots+x_n$.

Recall that a category $\mathcal{C}$ is called **CMon**-enriched if we have a commutative monoid structure on each homset $\mathcal{C}(C, C')$ and function composition gives monoid homomorphisms $\mathcal{C}(C, C') \otimes \mathcal{C}(C', C'') \to \mathcal{C}(C, C'')$. Finite products in a category $\mathcal{C}$ are well-known to be biproducts (i.e. simultaneously products and coproducts) if and only if $\mathcal{C}$ is **CMon**-enriched (see e.g. [17]): define $[] \stackrel{\mathrm{def}}{=} 0$ and $[f, g] \stackrel{\mathrm{def}}{=} \pi_1; f + \pi_2; g$ and, conversely, $0 \stackrel{\mathrm{def}}{=} []$ and $f + g \stackrel{\mathrm{def}}{=} (\mathrm{id}, \mathrm{id}); [f, g]$.

### 5.2 Abstract Semantics

The language of §3 has a canonical interpretation in any Cartesian closed category $(\mathcal{C}, \mathbb{1}, \times, \Rightarrow)$, once we fix $\mathcal{C}$-objects $[\![\mathbf{real}^n]\!]$ to interpret $\mathbf{real}^n$ and $\mathcal{C}$-morphisms $[\![\mathsf{op}]\!] \in \mathcal{C}([\![\mathbf{Dom}(\mathsf{op})]\!], [\![\mathbf{real}^m]\!])$ to interpret $\mathsf{op} \in \mathsf{Op}^m_{n_1,\ldots,n_k}$. We interpret types $\tau$ and contexts $\Gamma$ as $\mathcal{C}$-objects $[\![\tau]\!]$ and $[\![\Gamma]\!]$: $[\![x_1 : \tau_1, \ldots, x_n : \tau_n]\!] \stackrel{\mathrm{def}}{=} [\![\tau_1]\!] \times \ldots \times [\![\tau_n]\!] \quad [\![\mathbf{1}]\!] \stackrel{\mathrm{def}}{=} \mathbb{1} \quad [\![\tau * \sigma]\!] \stackrel{\mathrm{def}}{=} [\![\tau]\!] \times [\![\sigma]\!] \quad [\![\tau \to \sigma]\!] \stackrel{\mathrm{def}}{=} [\![\tau]\!] \Rightarrow [\![\sigma]\!]$ .
We interpret terms $\Gamma \vdash t : \tau$ as morphisms $[\![t]\!]$ in $\mathcal{C}([\![\Gamma]\!], [\![\tau]\!])$:

$$[\![x_1 : \tau_1, \ldots, x_n : \tau_n \vdash x_k : \tau_k]\!] \stackrel{\mathrm{def}}{=} \pi_k \qquad [\![\langle\rangle]\!] \stackrel{\mathrm{def}}{=} () \qquad [\![\langle t, s\rangle]\!] \stackrel{\mathrm{def}}{=} ([\![t]\!], [\![s]\!])$$
$$[\![\mathbf{fst}\,]\!] \stackrel{\mathrm{def}}{=} \pi_1 \quad [\![\mathbf{snd}\,]\!] \stackrel{\mathrm{def}}{=} \pi_2 \qquad [\![\lambda x.t]\!] \stackrel{\mathrm{def}}{=} \Lambda([\![t]\!]) \qquad [\![t\, s]\!] \stackrel{\mathrm{def}}{=} ([\![t]\!], [\![s]\!]); \mathrm{ev}.$$

This is an instance of the universal property of **Syn** mentioned in §3.

We discuss how to extend $[\![-]\!]$ to apply to the full target language of §4. Suppose that $\mathcal{L} : \mathcal{C}^{op} \to \mathbf{Cat}$ is a locally indexed category (see e.g. [27, §§§9.3.4]), i.e. a (strict) contravariant functor from $\mathcal{C}$ to the category **Cat** of categories, such that $\mathrm{ob}\,\mathcal{L}(C) = \mathrm{ob}\,\mathcal{L}(C')$ and $\mathcal{L}(f)(L) = L$ for any object $L$ of $\mathrm{ob}\,\mathcal{L}(C)$ and any $f : C' \to C$ in $\mathcal{C}$. We say that $\mathcal{L}$ is *biadditive* if each category $\mathcal{L}(C)$ has (chosen) finite biproducts $(\mathbb{1}, \times)$ and $\mathcal{L}(f)$ preserves them, for any $f : C' \to C$ in $\mathcal{C}$, in the sense that $\mathcal{L}(f)(\mathbb{1}) = \mathbb{1}$ and $\mathcal{L}(f)(L \times L') = \mathcal{L}(f)(L) \times \mathcal{L}(f)(L')$. We say that it *supports* $!(-) \otimes (-)$*-types and* $\Rightarrow$*-types*, if $\mathcal{L}(\pi_1)$ has a left adjoint $!C' \otimes_C -$ and a right adjoint functor $C' \Rightarrow_C -$, for each product projection $\pi_1 : C \times C' \to C$ in $\mathcal{C}$, satisfying a Beck-Chevalley condition: $!C' \otimes_C L = !C' \otimes_{C''} L$ and $C' \Rightarrow_C L = C' \Rightarrow_{C''} L$ for any $C, C'' \in \mathrm{ob}\,\mathcal{C}$. We simply write $!C' \otimes L$ and $C' \Rightarrow L$. Let us write

$\Phi$ and $\Psi$ for the natural isomorphisms $\mathcal{L}(C)(!C' \otimes L, L') \xrightarrow{\cong} \mathcal{L}(C \times C')(L, L')$ and $\mathcal{L}(C \times C)(L, L') \xrightarrow{\cong} \mathcal{L}(C)(L, C' \Rightarrow L')$. We say that $\mathcal{L}$ *supports Cartesian* $\multimap$*-types* if the functor $\mathcal{C}^{op} \to \mathbf{Set}$; $C \mapsto \mathcal{L}(C)(L, L')$ is representable for any objects $L, L'$ of $\mathcal{L}$. That is, we have objects $L \multimap L'$ of $\mathcal{C}$ with isomorphisms $\underline{\Lambda} : \mathcal{L}(C)(L, L') \xrightarrow{\cong} \mathcal{C}(C, L \multimap L')$, natural in $C$. We call an $\mathcal{L}$ satisfying all these conditions a *categorical model* of the language of §4. In particular, any biadditive model of intuitionistic linear logic [29,17] is such a categorical model.

If we choose $[\![\underline{\mathbf{real}}^n]\!] \in \mathrm{ob}\,\mathcal{L}$ to interpret $\underline{\mathbf{real}}^n$ and compatible $\mathcal{L}$-morphisms $[\![\mathsf{lop}]\!]$ in $\mathcal{L}([\![\mathbf{Dom}(\mathsf{lop})]\!])([\![\mathbf{LDom}(\mathsf{lop})]\!], [\![\underline{\mathbf{real}}^k]\!])$ for each $\mathsf{LOp}^m_{n_1,\ldots,n_k;n'_1,\ldots,n'_l}$, then we can interpret linear types $\underline{\tau}$ as objects $[\![\underline{\tau}]\!]$ of $\mathcal{L}$:

$$[\![\underline{\mathbf{1}}]\!] \overset{\mathrm{def}}{=} \mathbb{1} \quad [\![\underline{\tau} * \underline{\sigma}]\!] \overset{\mathrm{def}}{=} [\![\underline{\tau}]\!] \times [\![\underline{\sigma}]\!] \quad [\![\tau \to \underline{\sigma}]\!] \overset{\mathrm{def}}{=} [\![\tau]\!] \Rightarrow [\![\underline{\sigma}]\!] \quad [\![!\tau \otimes \underline{\sigma}]\!] \overset{\mathrm{def}}{=} ![\![\tau]\!] \otimes [\![\underline{\sigma}]\!].$$

We can interpret $\underline{\tau} \multimap \underline{\sigma}$ as the $\mathcal{C}$-object $[\![\underline{\tau} \multimap \underline{\sigma}]\!] \overset{\mathrm{def}}{=} [\![\underline{\tau}]\!] \multimap [\![\underline{\sigma}]\!]$. Finally, we can interpret terms $\Gamma \vdash t : \tau$ as morphisms $[\![t]\!]$ in $\mathcal{C}([\![\Gamma]\!], [\![\tau]\!])$ and terms $\Gamma; x : \underline{\tau} \vdash t : \underline{\sigma}$ as $[\![t]\!]$ in $\mathcal{L}([\![\Gamma]\!])([\![\underline{\tau}]\!], [\![\underline{\sigma}]\!])$:

$$[\![\Gamma; x : \underline{\tau} \vdash x : \underline{\tau}]\!] \overset{\mathrm{def}}{=} \mathrm{id}_{[\![\underline{\tau}]\!]} \quad [\![\langle\rangle]\!] \overset{\mathrm{def}}{=} () \quad [\![\langle t, s\rangle]\!] \overset{\mathrm{def}}{=} ([\![t]\!], [\![s]\!]) \quad [\![\mathbf{fst}\,]\!] \overset{\mathrm{def}}{=} \pi_1 \quad [\![\mathbf{snd}\,]\!] \overset{\mathrm{def}}{=} \pi_2$$

$$[\![\lambda x.t]\!] \overset{\mathrm{def}}{=} \Psi([\![t]\!]) \quad [\![t\, s]\!] \overset{\mathrm{def}}{=} \mathcal{L}((\mathrm{id}, [\![s]\!]))(\Psi^{-1}([\![t]\!]))$$

$$[\![!t \otimes s]\!] \overset{\mathrm{def}}{=} \mathcal{L}((\mathrm{id}, [\![t]\!]))(\Phi(\mathrm{id})); (![\![\sigma]\!] \otimes [\![s]\!]) \quad [\![\mathbf{case}\, t\, \mathbf{of}\, !y \otimes x \to s]\!] \overset{\mathrm{def}}{=} [\![t]\!]; \Phi^{-1}([\![s]\!])$$

$$[\![\underline{\lambda} x.t]\!] \overset{\mathrm{def}}{=} \underline{\Lambda}([\![t]\!]) \quad [\![t\{s\}]\!] \overset{\mathrm{def}}{=} \underline{\Lambda}^{-1}([\![t]\!]); [\![s]\!] \quad [\![\underline{0}]\!] \overset{\mathrm{def}}{=} [] \quad [\![t + s]\!] \overset{\mathrm{def}}{=} (\mathrm{id}, \mathrm{id}); [[\![t]\!], [\![s]\!]].$$

Observe that we interpret $\underline{0}$ and $+$ using the biproduct structure of $\mathcal{L}$.

**Proposition 1.** *The interpretation* $[\![-]\!]$ *of the language of* §4 *in categorical models is both sound and complete with respect to the* $\beta\eta+$*-equational theory:* $t \overset{\beta\eta+}{=} s$ *iff* $[\![t]\!] = [\![s]\!]$ *in each such model.*

Soundness follows by case analysis on the $\beta\eta+$-rules. Completeness follows by the construction of the syntactic model $\mathbf{LSyn} : \mathbf{CSyn}^{op} \to \mathbf{Cat}$:

- **CSyn** extends its full subcategory **Syn** with Cartesian $\multimap$-types;
- Objects of $\mathbf{LSyn}(\tau)$ are linear types $\underline{\sigma}$ of our target language.
- Morphisms in $\mathbf{LSyn}(\tau)(\underline{\sigma}, \underline{\rho})$ are terms $x : \tau; y : \underline{\sigma} \vdash t : \underline{\rho}$ modulo $(\alpha)\beta\eta+$-equivalence.
- Identities in $\mathbf{LSyn}(\tau)$ are represented by the terms $x : \tau; y : \underline{\sigma} \vdash y : \underline{\sigma}$.
- Composition of $x : \tau; y_1 : \underline{\sigma}_1 \vdash t : \underline{\sigma}_2$ and $x : \tau; y_2 : \underline{\sigma}_2 \vdash t : \underline{\sigma}_3$ in $\mathbf{LSyn}(\tau)$ is defined by the capture avoiding substitution $x : \tau; y_1 : \underline{\sigma}_1 \vdash s[{}^t/_{y_2}] : \underline{\sigma}_3$.
- Change of base $\mathbf{LSyn}(t) : \mathbf{LSyn}(\tau) \to \mathbf{LSyn}(\tau')$ along $(x' : \tau' \vdash t : \tau) \in \mathbf{CSyn}(\tau', \tau)$ is defined $\mathbf{LSyn}(t)(x : \tau; y : \underline{\sigma} \vdash s : \underline{\rho}) \overset{\mathrm{def}}{=} x' : \tau'; y : \underline{\sigma} \vdash s[{}^t/_x] \;:\; \underline{\rho}$.
- All type formers are interpreted as one expects based on their notation, using introduction and elimination rules for the required structural isomorphisms.

#### 5.3 Concrete Semantics

**Diffeological Spaces** Throughout this paper, we have an instance of the abstract semantics of our languages in mind, as we intend to interpret $\mathbf{real}^n$ as the usual Euclidean space $\mathbb{R}^n$ and to interpret each program $x_1 : \mathbf{real}^{n_1}, \ldots, x_k : \mathbf{real}^{n_k} \vdash t : \mathbf{real}^m$ as a smooth ($C^\infty$-) function $\mathbb{R}^{n_1} \times \ldots \times \mathbb{R}^{n_k} \to \mathbb{R}^m$. A challenge is that the usual settings for multivariate calculus and differential geometry do not form Cartesian closed categories, obstructing the interpretation of higher types (see [20, Appx. A]). A solution, recently employed by [20], is to work with *diffeological spaces* [33,21], which generalise the usual notions of differentiability from Euclidean spaces and smooth manifolds to apply to higher types (as well as a range of other types such a sum and inductive types). We will also follow this route and use such spaces to construct our concrete semantics. Other valid options for a concrete semantics exist: convenient vector spaces [19,7], Frölicher spaces [18], or synthetic differential geometry [25], to name a few. We choose to work with diffeological spaces mostly because they seem to us to provide simplest way to define and analyse the semantics of a rich class of language features.

Diffeological spaces formalise the intuition that a higher-order function is smooth if it sends smooth functions to smooth functions, meaning that we can never use it to build non-smooth first-order functions. This intuition is reminiscent of a logical relation, and it is realised by *directly axiomatising smooth maps into the space*, rather than treating smoothness as a derived property.

**Definition 1.** *A* diffeological space $X = (|X|, \mathcal{P}_X)$ *consists of a set* $|X|$ *together with, for each* $n \in \mathbb{N}$ *and each open subset* $U$ *of* $\mathbb{R}^n$*, a set* $\mathcal{P}_X^U$ *of functions* $U \to |X|$ *called* plots*, such that*
- ***(constant)*** *all constant functions are plots;*
- ***(rearrangement)*** *if* $f : V \to U$ *is smooth and* $p \in \mathcal{P}_X^U$*, then* $f; p \in \mathcal{P}_X^V$*;*
- ***(gluing)*** *if* $\left(p_i \in \mathcal{P}_X^{U_i}\right)_{i \in I}$ *is a compatible family of plots* ($x \in U_i \cap U_j \Rightarrow p_i(x) = p_j(x)$) *and* $(U_i)_{i \in I}$ *covers* $U$*, then the gluing* $p : U \to |X| : x \in U_i \mapsto p_i(x)$ *is a plot.*

We think of plots as the maps that are axiomatically deemed “smooth”. We call a function $f : X \to Y$ between diffeological spaces *smooth* if, for all plots $p \in \mathcal{P}_X^U$, we have that $p; f \in \mathcal{P}_Y^U$. We write $\mathbf{Diff}(X, Y)$ for the set of smooth maps from $X$ to $Y$. Smooth functions compose, and so we have a category $\mathbf{Diff}$ of diffeological spaces and smooth functions. We give some examples of such spaces.

*Example 1 (Manifold diffeology).* Given any open subset $X$ of a Euclidean space $\mathbb{R}^n$ (or, more generally, a smooth manifold $X$), we can take the set of smooth ($C^\infty$) functions $U \to X$ in the traditional sense as $\mathcal{P}_X^U$. Given another such space $X'$, then $\mathbf{Diff}(X, X')$ coincides precisely with the set of smooth functions $X \to X'$ in the traditional sense of calculus and differential geometry.

Put differently, the categories $\mathbf{CartSp}$ of Euclidean spaces and $\mathbf{Man}$ of smooth manifolds with smooth functions form full subcategories of $\mathbf{Diff}$.

*Example 2 (Product diffeology).* Given diffeological spaces $(X_i)_{i \in I}$, we can equip $\prod_{i \in I} |X_i|$ with the *product diffeology*: $\mathcal{P}^U_{\prod_{i \in I} X_i} \stackrel{\text{def}}{=} \left\{ (\alpha_i)_{i \in I} \mid \alpha_i \in \mathcal{P}^U_{X_i} \right\}$.

*Example 3 (Functional diffeology).* Given diffeological spaces $X, Y$, we can equip $\mathbf{Diff}(X, Y)$ with the *functional diffeology* $\mathcal{P}^U_{Y^X} \stackrel{\text{def}}{=} \{ \Lambda(\alpha) \mid \alpha \in \mathbf{Diff}(U \times X, Y) \}$.

Examples 2 and 3 give us the categorical product and exponential objects, respectively, in **Diff**. The embeddings of **CartSp** and **Man** into **Diff** preserve products (and coproducts).

We work with the concrete semantics, where we fix $\mathcal{C} = \mathbf{Diff}$ as the target for interpreting Cartesian types and their terms. That is, by choosing the interpretation $[\![\mathbf{real}^n]\!] \stackrel{\text{def}}{=} \mathbb{R}^n$, and by interpreting each $\mathsf{op} \in \mathsf{Op}^m_{n_1,\ldots,n_k}$ as the smooth function $[\![\mathsf{op}]\!] : \mathbb{R}^{n_1} \times \ldots \times \mathbb{R}^{n_k} \to \mathbb{R}^m$ that it is intended to represent, we obtain a unique interpretation $[\![-]\!] : \mathbf{CSyn} \to \mathbf{Diff}$.

**Diffeological Monoids** To interpret linear types and their terms, we need a semantic setting $\mathcal{L}$ that is both compatible with **Diff** and enriched over the category of commutative monoids. We choose to work with *commutative diffeological monoids*. That is, commutative monoids internal to the category **Diff**.

**Definition 2.** *A* diffeological monoid $X = (|X|, \mathcal{P}_X, 0_X, +_X)$ *consists of a diffeological space* $(|X|, \mathcal{P}_X)$ *with a monoid structure* $(0_X \in |X|, (+_X) : |X| \times |X| \to |X|)$*, such that* $+_X$ *is smooth. We call a diffeological monoid* commutative *if the underlying monoid structure on* $|X|$ *is commutative.*

We write $\mathbf{Diff_{CM}}$ for the category whose objects are commutative diffeological monoids and whose morphisms $(|X|, \mathcal{P}_X, 0_X, +_X) \to (|Y|, \mathcal{P}_Y, 0_Y, +_Y)$ are functions $f : |X| \to |Y|$ that are both smooth $(|X|, \mathcal{P}_X) \to (|Y|, \mathcal{P}_Y)$ and monoid homomorphisms $(|X|, 0_X, +_X) \to (|Y|, 0_Y, +_Y)$. Given that $\mathbf{Diff_{CM}}$ is **CMon**-enriched, finite products are biproducts.

*Example 4.* The real numbers $\mathbb{R}$ form a commutative diffeological monoid $\underline{\mathbb{R}}$ by combining its standard diffeology with its usual commutative monoid structure $(0, +)$. Similarly, $\underline{\mathbb{N}} \in \mathbf{Diff_{CM}}$ by equipping $\mathbb{N}$ with $(0, +)$ and the discrete diffeology, in which plots are locally constant functions.

*Example 5.* We form the (categorical) product in $\mathbf{Diff_{CM}}$ of $(X_i)_{i \in I}$ by equipping $\prod_{i \in I} |X_i|$ with the product diffeology and product monoid structure.

*Example 6.* For a commutative diffeological monoid $X$, we can equip the monoid $!(|X|, 0_X, +_X)$ with the diffeology $\mathcal{P}^U_{!X} \stackrel{\text{def}}{=} \{\sum_{i=1}^n \alpha_i; \delta \mid n \in \mathbb{N} \text{ and } \alpha_i \in \mathcal{P}^U_X\}$.

*Example 7.* Given commutative diffeological monoids $X$ and $Y$, we can equip the tensor product monoid $(|X|, 0_X, +_X) \otimes (|Y|, 0_Y, +_Y)$ with the *tensor product diffeology*: $\mathcal{P}^U_{X \otimes Y} \stackrel{\text{def}}{=} \{\sum_{i=1}^n \alpha_i \otimes \beta_i \mid n \in \mathbb{N} \text{ and } \alpha_i \in \mathcal{P}^U_X, \beta_i \in \mathcal{P}^U_Y\}$.

In this paper, we only use the combined operation $!X \otimes Y$ (read: $(!X) \otimes Y$).

*Example 8.* Given commutative diffeological monoids $X$ and $Y$, we can define a commutative diffeological monoid $X \multimap Y$ with underlying set $\mathbf{Diff_{CM}}(X, Y)$, $0_{X \multimap Y}(x) \stackrel{\text{def}}{=} 0_Y$, $(f +_{X \multimap Y} g)(x) \stackrel{\text{def}}{=} f(x) +_Y g(x)$ and
$\mathcal{P}^U_{X \multimap Y} \stackrel{\text{def}}{=} \left\{\alpha : U \to |X \multimap Y| \mid \alpha \in \mathcal{P}^U_{(|X|, \mathcal{P}_X) \Rightarrow (|Y|, \mathcal{P}_Y)}\right\}$.

In this paper, we will primarily be interested in $X \multimap Y$ as a diffeological space, and we will mostly disregard its monoid structure for now.

*Example 9.* Given a diffeological space $X$ and a commutative diffeological monoid $Y$, we can define a commutative diffeological monoid structure $X \Rightarrow Y$ on $X \Rightarrow (|Y|, \mathcal{P}_Y)$ by using the pointwise monoid structure: $0_{X\Rightarrow Y}(x) \stackrel{\text{def}}{=} 0_Y$ and $(f +_{X\Rightarrow Y} g)(x) \stackrel{\text{def}}{=} f(x) +_Y g(x)$.

Given $f \in \mathbf{Diff}(X, Y)$, we can define $!f \in \mathbf{Diff_{CM}}(!X, !Y)$ by $!f(\sum_{i=1}^n x) = \sum_{i=1}^n f(x)$. ! is a left adjoint to the obvious forgetful functor $\mathbf{Diff_{CM}} \to \mathbf{Diff}$, while $!(X \times Y) \cong !X \otimes !Y$ and $!\mathbb{1} \cong \mathbb{N}$. Seeing that $(\mathbb{N}, \otimes, \multimap)$ defines a symmetric monoidal closed structure on $\mathbf{Diff_{CM}}$, cognoscenti will recognise that $(\mathbf{Diff}, \mathbb{1}, \times, \Rightarrow) \leftrightarrows (\mathbf{Diff_{CM}}, \mathbb{N}, \mathbb{1}, \times, \otimes, \multimap)$ is a model of intuitionistic linear logic [29]. In fact, seeing that $\mathbf{Diff_{CM}}$ is **CMon**-enriched, the model is biadditive [17].

However, we do not need such a rich type system. For us, the following suffices. Define $\mathbf{Diff_{CM}}(X)$, for $X \in \mathrm{ob}\,\mathbf{Diff}$, to have the objects of $\mathbf{Diff_{CM}}$ and homsets $\mathbf{Diff_{CM}}(X)(Y, Z) \stackrel{\text{def}}{=} \mathbf{Diff}(X, Y \multimap Z)$. Identities and composition are defined as $x \mapsto (y \mapsto y)$ and $f;_{\mathbf{Diff_{CM}}(X)} g$ is defined by $x \mapsto (f(x);_{\mathbf{Diff_{CM}}} g(x))$. Given $f \in \mathbf{Diff}(X, X')$, we define change-of-base $\mathbf{Diff_{CM}}(X') \to \mathbf{Diff_{CM}}(X)$ as $\mathbf{Diff_{CM}}(f)(g) \stackrel{\text{def}}{=} f;_{\mathbf{Diff}} g$. $\mathbf{Diff_{CM}}(-)$ defines a locally indexed category. By taking $\mathcal{C} = \mathbf{Diff}$ and $\mathcal{L}(-) = \mathbf{Diff_{CM}}(-)$, we obtain a concrete instance of our abstract semantics. Indeed, we have natural isomorphisms

$$\mathbf{Diff_{CM}}(X)(!X' \otimes Y, Z) \xrightarrow{\Phi} \mathbf{Diff_{CM}}(X \times X')(Y, Z)$$

$$\mathbf{Diff_{CM}}(X \times X')(Y, Z) \xrightarrow{\Psi} \mathbf{Diff_{CM}}(X)(Y, X' \Rightarrow Z)$$

$$\Phi(f)(x, x')(y) \stackrel{\text{def}}{=} f(x)(\delta(x') \otimes y) \quad \Phi^{-1}(f)(x)(\sum_{i=1}^n (\delta(x'_i) \otimes y_i)) \stackrel{\text{def}}{=} \sum_{i=1}^n f(x, x'_i)(y_i)$$
$$\Psi(f)(x)(y)(x') \stackrel{\text{def}}{=} f(x, x')(y) \qquad \Psi^{-1}(f)(x, x')(y) \stackrel{\text{def}}{=} f(x)(y)(x').$$

The prime motivating examples of morphisms in this category are derivatives. Recall that the *derivative at* $x$, $Df(x)$, and *transposed derivative at* $x$, $(Df)^t(x)$, of a smooth function $f : \mathbb{R}^n \to \mathbb{R}^m$ are defined as the unique functions $Df(x) : \mathbb{R}^n \to \mathbb{R}^m$ and $(Df)^t(x) : \mathbb{R}^m \to \mathbb{R}^n$ satisfying

$$Df(x)(v) = \lim_{\delta \to 0} \frac{f(x + \delta \cdot v) - f(x)}{\delta} \qquad (Df)^t(x)(w) \bullet v = w \bullet Df(x)(v),$$

where we write $v \bullet v'$ for the inner product $\sum_{i=1}^n (\pi_i v) \cdot (\pi_i v')$ of vectors $v, v' \in \mathbb{R}^n$. Now, for $f \in \mathbf{Diff}(\mathbb{R}^n, \mathbb{R}^m)$, $Df$ and $(Df)^t$ give maps in $\mathbf{Diff_{CM}}(\mathbb{R}^n)(\underline{\mathbb{R}}^n, \underline{\mathbb{R}}^m)$ and $\mathbf{Diff_{CM}}(\mathbb{R}^n)(\underline{\mathbb{R}}^m, \underline{\mathbb{R}}^n)$, respectively. Indeed, derivatives $Df(x)$ of $f$ at $x$ are linear functions, as are transposed derivatives $(Df)^t(x)$. Both depend smoothly on $x$ in case $f$ is $C^\infty$-smooth. Note that the derivatives are not merely linear in the sense of preserving 0 and +. They are also multiplicative in the sense that $(Df)(x)(c \cdot v) = c \cdot (Df)(x)(v)$. *We could have captured this property by working with vector spaces internal to* **Diff**. *However, we will not need this property to*

*phrase or establish correctness of AD.* Therefore, we restrict our attention to the more straightforward structure of commutative monoids.

Defining $[\![\underline{\mathbf{real}}^n]\!] \stackrel{\text{def}}{=} \underline{\mathbb{R}}^n$ and interpreting each $\mathsf{lop} \in \mathsf{LOp}$ as the smooth function $[\![\mathsf{lop}]\!] : (\mathbb{R}^{n_1} \times \ldots \times \mathbb{R}^{n_k}) \to (\underline{\mathbb{R}}^{n'_1} \times \ldots \times \underline{\mathbb{R}}^{n'_l}) \multimap \underline{\mathbb{R}}^m$ it is intended to represent, we obtain a canonical interpretation of our target language in $\mathbf{Diff_{CM}}$.

## 6 Pairing Primals with Tangents/Adjoints, Categorically

In this section, we show that any categorical model $\mathcal{L} : \mathcal{C}^{op} \to \mathbf{Cat}$ of our target language gives rise to two Cartesian closed categories $\Sigma_{\mathcal{C}}\mathcal{L}$ and $\Sigma_{\mathcal{C}}\mathcal{L}^{op}$ (which we wrote $\overrightarrow{\mathfrak{D}}[\mathcal{L}]$ and $\overleftarrow{\mathfrak{D}}[\mathcal{L}]$ in §2). We believe these observations of Cartesian closure are novel. Surprisingly, they are highly relevant for obtaining a principled understanding of AD on a higher-order language: the former for forward AD, and the latter for reverse AD. Applying these constructions to the syntactic category $\mathbf{LSyn} : \mathbf{CSyn}^{op} \to \mathbf{Cat}$ of our language, we produce a canonical definition of the AD macros, as the canonical interpretation of the λ-calculus in the Cartesian closed categories $\Sigma_{\mathbf{CSyn}}\mathbf{LSyn}$ and $\Sigma_{\mathbf{CSyn}}\mathbf{LSyn}^{op}$. In addition, when we apply this construction to the denotational semantics $\mathbf{Diff_{CM}} : \mathbf{Diff}^{op} \to \mathbf{Cat}$ and invoke a categorical logical relations technique, known as *subsconing*, we find an elegant correctness proof of the source code transformations. The abstract construction delineated in this section is in many ways the theoretical crux of this paper.

### 6.1 Grothendieck Constructions on Strictly Indexed Categories

Recall that for any strictly indexed category, i.e. a (strict) functor $\mathcal{L} : \mathcal{C}^{op} \to \mathbf{Cat}$, we can consider its total category (or Grothendieck construction) $\Sigma_{\mathcal{C}}\mathcal{L}$, which is a fibred category over $\mathcal{C}$ (see [23, sections A1.1.7, B1.3.1]). We can view it as a $\Sigma$-type of categories, which generalizes the Cartesian product. Concretely, its objects are pairs $(A_1, A_2)$ of objects $A_1$ of $\mathcal{C}$ and $A_2$ of $\mathcal{L}(A_1)$. Its morphisms $(A_1, A_2) \to (B_1, B_2)$ are pairs $(f_1, f_2)$ of a morphism $f_1 : A_1 \to B_1$ in $\mathcal{C}$ and a morphism $f_2 : A_2 \to \mathcal{L}(f_1)(B_2)$ in $\mathcal{L}(A_1)$. Identities are $\mathrm{id}_{(A_1,A_2)} \stackrel{\text{def}}{=} (\mathrm{id}_{A_1}, \mathrm{id}_{A_2})$ and composition is $(f_1, f_2); (g_1, g_2) \stackrel{\text{def}}{=} (f_1; g_1, f_2; \mathcal{L}(f_1)(g_2))$. Further, given a strictly indexed category $\mathcal{L} : \mathcal{C}^{op} \to \mathbf{Cat}$, we can consider its fibrewise dual category $\mathcal{L}^{op} : \mathcal{C}^{op} \to \mathbf{Cat}$, which is defined as the composition $\mathcal{C}^{op} \xrightarrow{\mathcal{L}} \mathbf{Cat} \xrightarrow{op} \mathbf{Cat}$. Thus, we can apply the same construction to $\mathcal{L}^{op}$ to obtain a category $\Sigma_{\mathcal{C}}\mathcal{L}^{op}$.

### 6.2 Structure of $\Sigma_{\mathcal{C}}\mathcal{L}$ and $\Sigma_{\mathcal{C}}\mathcal{L}^{op}$ for Locally Indexed Categories

§§6.1 applies, in particular, to the locally indexed categories of §5. In this case, we will analyze the categorical structure of $\Sigma_{\mathcal{C}}\mathcal{L}$ and $\Sigma_{\mathcal{C}}\mathcal{L}^{op}$. For reference, we first give a concrete description.

$\Sigma_{\mathcal{C}}\mathcal{L}$ is the following category:

- objects are pairs $(A_1, A_2)$ of objects $A_1$ of $\mathcal{C}$ and $A_2$ of $\mathcal{L}$;

- morphisms $(A_1, A_2) \to (B_1, B_2)$ are pairs $(f_1, f_2)$ with $f_1 : A_1 \to B_1 \in \mathcal{C}$ and $f_2 : A_2 \to B_2 \in \mathcal{L}(A_1)$;
- composition of $(A_1, A_2) \xrightarrow{(f_1,f_2)} (B_1, B_2)$ and $(B_1, B_2) \xrightarrow{(g_1,g_2)} (C_1, C_2)$ is given by $(f_1; g_1, f_2; \mathcal{L}(f_1)(g_2))$ and identities $\mathrm{id}_{(A_1,A_2)}$ are $(\mathrm{id}_{A_1}, \mathrm{id}_{A_2})$.

$\Sigma_{\mathcal{C}}\mathcal{L}^{op}$ is the following category:

- objects are pairs $(A_1, A_2)$ of objects $A_1$ of $\mathcal{C}$ and $A_2$ of $\mathcal{L}$;
- morphisms $(A_1, A_2) \to (B_1, B_2)$ are pairs $(f_1, f_2)$ with $f_1 : A_1 \to B_1 \in \mathcal{C}$ and $f_2 : B_2 \to A_2 \in \mathcal{L}(A_1)$;
- composition of $(A_1, A_2) \xrightarrow{(f_1,f_2)} (B_1, B_2)$ and $(B_1, B_2) \xrightarrow{(g_1,g_2)} (C_1, C_2)$ is given by $(f_1; g_1, \mathcal{L}(f_1)(g_2); f_2)$ and identities $\mathrm{id}_{(A_1,A_2)}$ are $(\mathrm{id}_{A_1}, \mathrm{id}_{A_2})$.

We examine the categorical structure present in $\Sigma_{\mathcal{C}}\mathcal{L}$ and $\Sigma_{\mathcal{C}}\mathcal{L}^{op}$ for categorical models $\mathcal{L}$ in the sense of §5 (i.e., in case $\mathcal{L}$ has biproducts and supports $\Rightarrow$-, $!(-)\otimes(-)$-, and Cartesian $\multimap$-types). We believe this is a novel observation. We will make heavy use of it to define our AD algorithms and to prove them correct.

**Proposition 2.** *$\Sigma_{\mathcal{C}}\mathcal{L}$ has terminal object $\mathbb{1} = (\mathbb{1}, \mathbb{1})$, binary product $(A_1, A_2) \times (B_1, B_2) = (A_1 \times B_1, A_2 \times B_2)$, and exponential $(A_1, A_2) \Rightarrow (B_1, B_2) = (A_1 \Rightarrow (B_1 \times (A_2 \multimap B_2)), A_1 \Rightarrow B_2)$.*

*Proof.* We have (natural) bijections

$$\Sigma_{\mathcal{C}}\mathcal{L}((A_1, A_2), (\mathbb{1}, \mathbb{1})) = \mathcal{C}(A_1, \mathbb{1}) \times \mathcal{L}(A_1)(A_2, \mathbb{1}) \cong \mathbb{1} \times \mathbb{1} \cong \mathbb{1} \qquad \{ \ \mathbb{1} \text{ terminal in } \mathcal{C} \text{ and } \mathcal{L}(A_1) \ \}$$

$$\begin{aligned}
&\Sigma_{\mathcal{C}}\mathcal{L}((A_1, A_2), (B_1 \times C_1, B_2 \times C_2)) = \mathcal{C}(A_1, B_1 \times C_1) \times \mathcal{L}(A_1)(A_2, B_2 \times C_2) \\
&\cong \mathcal{C}(A_1, B_1) \times \mathcal{C}(A_1, C_1) \times \mathcal{L}(A_1)(A_2, B_2) \times \mathcal{L}(A_1)(A_2, C_2) && \{ \ \times \text{ product in } \mathcal{C} \text{ and } \mathcal{L}(A_1) \ \} \\
&\cong \Sigma_{\mathcal{C}}\mathcal{L}((A_1, A_2), (B_1, B_2)) \times \Sigma_{\mathcal{C}}\mathcal{L}((A_1, A_2), (C_1, C_2))
\end{aligned}$$

$$\begin{aligned}
&\Sigma_{\mathcal{C}}\mathcal{L}((A_1, A_2) \times (B_1, B_2), (C_1, C_2)) = \Sigma_{\mathcal{C}}\mathcal{L}((A_1 \times B_1, A_2 \times B_2), (C_1, C_2)) \\
&= \mathcal{C}(A_1 \times B_1, C_1) \times \mathcal{L}(A_1 \times B_1)(A_2 \times B_2, C_2) \\
&\cong \mathcal{C}(A_1 \times B_1, C_1) \times \mathcal{L}(A_1 \times B_1)(A_2, C_2) \times \mathcal{L}(A_1 \times B_1)(B_2, C_2) && \{ \ \times \text{ coproducts in } \mathcal{L}(A_1 \times B_1) \ \} \\
&\cong \mathcal{C}(A_1 \times B_1, C_1) \times \mathcal{L}(A_1)(A_2, B_1 \Rightarrow C_2) \times \mathcal{L}(A_1 \times B_1)(B_2, C_2) && \{ \ \Rightarrow\text{-types in } \mathcal{L} \ \} \\
&\cong \mathcal{C}(A_1 \times B_1, C_1) \times \mathcal{L}(A_1)(A_2, B_1 \Rightarrow C_2) \times \mathcal{C}(A_1 \times B_1, B_2 \multimap C_2) && \{ \ \text{Cartesian } \multimap\text{-types} \ \} \\
&\cong \mathcal{C}(A_1 \times B_1, C_1 \times (B_2 \multimap C_2)) \times \mathcal{L}(A_1)(A_2, B_1 \Rightarrow C_2) && \{ \ \times \text{ is product in } \mathcal{C} \ \} \\
&\cong \mathcal{C}(A_1, B_1 \Rightarrow (C_1 \times (B_2 \multimap C_2))) \times \mathcal{L}(A_1)(A_2, B_1 \Rightarrow C_2) && \{ \ \Rightarrow \text{ is exponential in } \mathcal{C} \ \} \\
&= \Sigma_{\mathcal{C}}\mathcal{L}((A_1, A_2), (B_1 \Rightarrow (C_1 \times (B_2 \multimap C_2)), B_1 \Rightarrow C_2)) \\
&= \Sigma_{\mathcal{C}}\mathcal{L}((A_1, A_2), (B_1, B_2) \Rightarrow (C_1, C_2)).
\end{aligned}$$

□

We observe that we need $\mathcal{L}$ to have biproducts (equivalently: to be **CMon** enriched) in order to show Cartesian closure. Further, we need linear $\Rightarrow$-types and Cartesian $\multimap$-types to construct exponentials.

**Proposition 3.** *$\Sigma_{\mathcal{C}}\mathcal{L}^{op}$ has terminal object $\mathbb{1} = (\mathbb{1}, \mathbb{1})$, binary product $(A_1, A_2) \times (B_1, B_2) = (A_1 \times B_1, A_2 \times B_2)$, and exponential $(A_1, A_2) \Rightarrow (B_1, B_2) = (A_1 \Rightarrow (B_1 \times (B_2 \multimap A_2)), !A_1 \otimes B_2)$.*

*Proof.* We have (natural) bijections

$$\Sigma_{\mathcal{C}}\mathcal{L}^{op}((A_1,A_2),(\mathbb{1},\mathbb{1})) = \mathcal{C}(A_1,\mathbb{1}) \times \mathcal{L}(A_1)(\mathbb{1},A_2) \cong \mathbb{1}\times\mathbb{1} \cong \mathbb{1} \qquad \{\ \mathbb{1} \text{ terminal in } \mathcal{C}, \text{ initial in } \mathcal{L}(A_1)\ \}$$

$$\begin{aligned}
&\Sigma_{\mathcal{C}}\mathcal{L}^{op}((A_1,A_2),(B_1\times C_1,B_2\times C_2)) = \mathcal{C}(A_1,B_1\times C_1)\times\mathcal{L}(A_1)(B_2\times C_2,A_2)\\
&\cong \mathcal{C}(A_1,B_1)\times\mathcal{C}(A_1,C_1)\times\mathcal{L}(A_1)(B_2,A_2)\times\mathcal{L}(A_1)(C_2,A_2) && \{\ \times \text{ product in } \mathcal{C}, \text{ coproduct in } \mathcal{L}(A_1)\ \}\\
&= \Sigma_{\mathcal{C}}\mathcal{L}^{op}((A_1,A_2),(B_1,B_2))\times\Sigma_{\mathcal{C}}\mathcal{L}^{op}((A_1,A_2),(C_1,C_2))
\end{aligned}$$

$$\begin{aligned}
&\Sigma_{\mathcal{C}}\mathcal{L}^{op}((A_1,A_2)\times(B_1,B_2),(C_1,C_2)) = \Sigma_{\mathcal{C}}\mathcal{L}^{op}((A_1\times B_1,A_2\times B_2),(C_1,C_2))\\
&= \mathcal{C}(A_1\times B_1,C_1)\times\mathcal{L}(A_1\times B_1)(C_2,A_2\times B_2)\\
&\cong \mathcal{C}(A_1\times B_1,C_1)\times\mathcal{L}(A_1\times B_1)(C_2,A_2)\times\mathcal{L}(A_1\times B_1)(C_2,B_2) && \{\ \times \text{ is product in } \mathcal{L}(A_1\times B_1)\ \}\\
&\cong \mathcal{C}(A_1\times B_1,C_1)\times\mathcal{C}(A_1\times B_1,C_2\multimap B_2)\times\mathcal{L}(A_1\times B_1)(C_2,A_2) && \{\ \text{Cartesian } \multimap\text{-types}\ \}\\
&\cong \mathcal{C}(A_1\times B_1,C_1\times(C_2\multimap B_2))\times\mathcal{L}(A_1\times B_1)(C_2,A_2) && \{\ \times \text{ is product in } \mathcal{C}\ \}\\
&\cong \mathcal{C}(A_1,B_1\Rightarrow(C_1\times(C_2\multimap B_2)))\times\mathcal{L}(A_1\times B_1)(C_2,A_2) && \{\ \Rightarrow \text{ is exponential in } \mathcal{C}\ \}\\
&\cong \mathcal{C}(A_1,B_1\Rightarrow(C_1\times(C_2\multimap B_2)))\times\mathcal{L}(A_1)(!B_1\otimes C_2,A_2) && \{\ !(-)\otimes(-)\text{-types}\ \}\\
&= \Sigma_{\mathcal{C}}\mathcal{L}^{op}((A_1,A_2),(B_1\Rightarrow(C_1\times(C_2\multimap B_2)),!B_1\otimes C_2))\\
&= \Sigma_{\mathcal{C}}\mathcal{L}^{op}((A_1,A_2),(B_1,B_2)\Rightarrow(C_1,C_2)).
\end{aligned}$$

□

Observe that we need the biproduct structure of $\mathcal{L}$ to construct finite products in $\Sigma_{\mathcal{C}}\mathcal{L}^{op}$. Further, we need Cartesian $\multimap$-types and $!(-)\otimes(-)$-types, but not biproducts, to construct exponentials.

## 7 Novel AD Algorithms as Source-Code Transformations

As $\Sigma_{\mathbf{CSyn}}\mathbf{LSyn}$ and $\Sigma_{\mathbf{CSyn}}\mathbf{LSyn}^{op}$ are both Cartesian closed categories by §6, the universal property of **Syn** yields unique structure-preserving macros, $\overrightarrow{\mathcal{D}}(-) : \mathbf{Syn} \to \Sigma_{\mathbf{CSyn}}\mathbf{LSyn}$ (forward AD) and $\overleftarrow{\mathcal{D}}(-) : \mathbf{Syn} \to \Sigma_{\mathbf{CSyn}}\mathbf{LSyn}^{op}$ (reverse AD), once we fix a compatible definition for the macros on $\mathbf{real}^n$ and basic operations $\mathsf{op}$. By definition of equality in **Syn**, $\Sigma_{\mathbf{CSyn}}\mathbf{LSyn}$ and $\Sigma_{\mathbf{CSyn}}\mathbf{LSyn}^{op}$, *these macros automatically respect equational reasoning principles*, in the sense that $t \stackrel{\beta\eta}{=} s$ implies that $\overrightarrow{\mathcal{D}}(t) \stackrel{\beta\eta+}{=} \overrightarrow{\mathcal{D}}(s)$ and $\overleftarrow{\mathcal{D}}(t) \stackrel{\beta\eta+}{=} \overleftarrow{\mathcal{D}}(s)$.

We need to choose suitable terms $D\mathsf{op}(x;y)$ and $D\mathsf{op}^t(x;y)$ to represent the forward- and reverse-mode derivatives of the basic operations $\mathsf{op}\in\mathsf{Op}^m_{n_1,\ldots,n_k}$. For example, for elementwise multiplication $(*)\in\mathsf{Op}^n_{n,n}$, we can define $D(*)(x;y) = (\mathbf{fst}\,x)*(\mathbf{snd}\,y)+(\mathbf{snd}\,x)*(\mathbf{fst}\,y)$ and $D(*)^t(x;y) = \langle(\mathbf{snd}\,x)*y,(\mathbf{fst}\,x)*y\rangle$, where we use (linear) elementwise multiplication $(*)\in\mathsf{LOp}^n_{n;n}$. We represent derivatives as linear functions. This representation allows for efficient Jacobian-vector/adjoint product implementations, which avoid first calculating a full Jacobian and next taking a product. Such implementations are known to be important to achieve performant AD systems.

$$\overrightarrow{\mathcal{D}}(\mathbf{real}^n)_1 \stackrel{\text{def}}{=} \mathbf{real}^n \quad \overrightarrow{\mathcal{D}}(\mathbf{real}^n)_2 \stackrel{\text{def}}{=} \underline{\mathbf{real}}^n \quad \overleftarrow{\mathcal{D}}(\mathbf{real}^n)_1 \stackrel{\text{def}}{=} \mathbf{real}^n \quad \overrightarrow{\mathcal{D}}(\mathbf{real}^n)_2 \stackrel{\text{def}}{=} \underline{\mathbf{real}}^n$$

$$\overrightarrow{\mathcal{D}}(\mathsf{op})_1 \stackrel{\text{def}}{=} \mathsf{op} \quad \overrightarrow{\mathcal{D}}(\mathsf{op})_2 \stackrel{\text{def}}{=} x : \mathbf{real}^{n_1}*..*\mathbf{real}^{n_k}; y : \underline{\mathbf{real}}^{n_1}*..*\underline{\mathbf{real}}^{n_k} \vdash D\mathsf{op}(x;y) : \underline{\mathbf{real}}^m$$

$$\overleftarrow{\mathcal{D}}(\mathsf{op})_1 \stackrel{\text{def}}{=} \mathsf{op} \quad \overleftarrow{\mathcal{D}}(\mathsf{op})_2 \stackrel{\text{def}}{=} x : \mathbf{real}^{n_1}*..*\mathbf{real}^{n_k}; y : \underline{\mathbf{real}}^m \vdash D\mathsf{op}^t(x;y) : \underline{\mathbf{real}}^{n_1}*..*\underline{\mathbf{real}}^{n_k}$$

For the AD transformations to be correct, it is important that these derivatives of language primitives are implemented correctly in the sense that

$$[\![x; y \vdash D\mathsf{op}(x; y)]\!] = D[\![\mathsf{op}]\!] \qquad [\![x; y \vdash D\mathsf{op}^t(x; y)]\!] = D[\![\mathsf{op}]\!]^t.$$

In practice, AD library developers tend to assume the subtle task of correctly implementing such derivatives $D\mathsf{op}(x; y)$ and $D\mathsf{op}^t(x; y)$ whenever a new primitive operation $\mathsf{op}$ is added to the library.

The extension of the AD macros $\overrightarrow{\mathcal{D}}$ and $\overleftarrow{\mathcal{D}}$ to the full source language are now canonically determined, as the unique Cartesian closed functors that extend the previous definitions, following the categorical structure described in §6. Because of the counter-intuitive nature of the Cartesian closed structures on $\Sigma_{\mathbf{CSyn}}\mathbf{LSyn}$ and $\Sigma_{\mathbf{CSyn}}\mathbf{LSyn}^{op}$, we list the full macros explicitly in [36, Appx. A].

## 8 Proving Reverse and Forward AD Semantically Correct

In this section, we will show that the source code transformations described in §7 correctly implement mathematical derivatives. We make correctness precise as the statement that for programs $x : \tau \vdash t : \sigma$ between first-order types $\tau$ and $\sigma$, i.e. types not containing any function type constructors, we have that $[\![\overrightarrow{\mathcal{D}}(t)_2]\!] = D[\![t]\!]$ and $[\![\overleftarrow{\mathcal{D}}(t)_2]\!] = (D[\![t]\!])^t$, where $[\![-]\!]$ is the semantics of §5. The proof mainly consists of logical relations arguments over the semantics in $\Sigma_{\mathbf{Diff}}\mathbf{Diff}_{\mathbf{CM}}$ and $\Sigma_{\mathbf{Diff}}\mathbf{Diff}_{\mathbf{CM}}{}^{op}$. This logical relations proof can be phrased in elementary terms, but the resulting argument is technical and would be hard to discover. Instead, we prefer to phrase it in terms of a categorical subsconing construction, a more abstract and elegant perspective on logical relations. We discovered the proof by taking this categorical perspective, and, while we have verified the elementary argument (see [36, Appx. D]), we would not otherwise have come up with it.

### 8.1 Preliminaries

**Subsconing** Logical relations arguments provide a powerful proof technique for demonstrating properties of typed programs. The arguments proceed by induction on the structure of types. Here, we briefly review the basics of categorical logical relations arguments, or *subsconing constructions*. We restrict to the level of generality that we need here, but we would like to point out that the theory applies much more generally.

Consider a Cartesian closed category $(\mathcal{C}, \mathbb{1}, \times, \Rightarrow)$. Suppose that we are given a functor $F : \mathcal{C} \to \mathbf{Set}$ to the category $\mathbf{Set}$ of sets and functions which preserves finite products in the sense that $F(\mathbb{1}) \cong \mathbb{1}$ and $F(C \times C') \cong F(C) \times F(C')$. Then, we can form the *subscone* of $F$, or category of logical relations over $F$, which is Cartesian closed, with a faithful Cartesian closed functor $\pi_1$ to $\mathcal{C}$ which forgets about the predicates [24]:

- objects are pairs $(C, P)$ of an object $C$ of $\mathcal{C}$ and a predicate $P \subseteq FC$;
- morphisms $(C, P) \to (C', P')$ are $\mathcal{C}$ morphisms $f : C \to C'$ which respect the predicates in the sense that $F(f)(P) \subseteq P'$;

– identities and composition are as in $\mathcal{C}$;
– $(\mathbb{1}, F\mathbb{1})$ is the terminal object, and products and exponentials are given by $(C,P)\times(C',P') = (C\times C', \{\alpha \in F(C\times C') \mid F(\pi_1)(\alpha)\in P, F(\pi_2)(\alpha)\in P'\})$ $(C,P)\Rightarrow(C',P') = (C\Rightarrow C', \{F(\pi_1)(\gamma) \mid \gamma\in F((C\Rightarrow C')\times C)$ s.t. $F(\pi_2)(\gamma)\in P$ implies $F(\mathrm{ev})(\gamma)\in P'\})$.

In typical applications, $\mathcal{C}$ can be the syntactic category of a language (like **Syn**), the codomain of a denotational semantics $[\![-]\!]$ (like **Diff**), or a product of the above, if we want to consider $n$-ary logical relations. Typically, $F$ tends to be a hom-functor (which always preserves products), like $\mathcal{C}(\mathbb{1},-)$ or $\mathcal{C}(C_0,-)$, for some important object $C_0$. When applied to the syntactic category **Syn** and $F = \mathbf{Syn}(\mathbf{1},-)$, the formulae for products and exponentials in the subscone clearly reproduce the usual recipes in traditional, syntactic logical relations arguments. As such, subsconing generalises standard logical relations methods.

### 8.2 Subsconing for Correctness of AD

We will apply the subsconing construction above to

$$\mathcal{C} = \mathbf{Diff}\times\Sigma_{\mathbf{Diff}}\mathbf{Diff}_{\mathbf{CM}}\quad F = \mathbf{Diff}\times\Sigma_{\mathbf{Diff}}\mathbf{Diff}_{\mathbf{CM}}((\mathbb{R},(\mathbb{R},\underline{\mathbb{R}})),-)\quad \text{(forward AD)}$$
$$\mathcal{C} = \mathbf{Diff}\times\Sigma_{\mathbf{Diff}}\mathbf{Diff}_{\mathbf{CM}}{}^{op}\; F = \mathbf{Diff}\times\Sigma_{\mathbf{Diff}}\mathbf{Diff}_{\mathbf{CM}}{}^{op}((\mathbb{R},(\mathbb{R},\underline{\mathbb{R}})),-)\; \text{(reverse AD)},$$

where we note that **Diff**, $\Sigma_{\mathbf{Diff}}\mathbf{Diff}_{\mathbf{CM}}$, and $\Sigma_{\mathbf{Diff}}\mathbf{Diff}_{\mathbf{CM}}{}^{op}$ are Cartesian closed (given the arguments of §5 and §6) and that the product of Cartesian closed categories is again Cartesian closed. Let us write $\overrightarrow{\mathbf{SScone}}$ and $\overleftarrow{\mathbf{SScone}}$, respectively, for the resulting categories of logical relations.

Seeing that $\overrightarrow{\mathbf{SScone}}$ and $\overleftarrow{\mathbf{SScone}}$ are Cartesian closed, we obtain unique Cartesian closed functors $(\!|-|\!)^f : \mathbf{Syn}\to\overrightarrow{\mathbf{SScone}}$ and $(\!|-|\!)^r : \mathbf{Syn}\to\overleftarrow{\mathbf{SScone}}$ once we fix an interpretation of $\mathbf{real}^n$ and all operations $\mathsf{op}$. We write $P^f_\tau$ and $P^r_\tau$, respectively, for the relations $\pi_2(\!|\tau|\!)^f$ and $\pi_2(\!|\tau|\!)^r$. Let us interpret

$$(\!|\mathbf{real}^n|\!)^f \overset{\text{def}}{=} (((\mathbb{R}^n,(\mathbb{R}^n,\underline{\mathbb{R}}^n)),\{(f,(g,h)) \mid f = g \text{ and } h = Df\}))$$
$$(\!|\mathbf{real}^n|\!)^r \overset{\text{def}}{=} (((\mathbb{R}^n,(\mathbb{R}^n,\underline{\mathbb{R}}^n)),\{(f,(g,h)) \mid f = g \text{ and } h = (Df)^t\}))$$
$$(\!|\mathsf{op}|\!)^f \overset{\text{def}}{=} ([\![\mathsf{op}]\!],([\![\overrightarrow{\mathcal{D}}(\mathsf{op})_1]\!],[\![\overrightarrow{\mathcal{D}}(\mathsf{op})_2]\!]))\qquad (\!|\mathsf{op}|\!)^r \overset{\text{def}}{=} ([\![\mathsf{op}]\!],([\![\overleftarrow{\mathcal{D}}(\mathsf{op})_1]\!],[\![\overleftarrow{\mathcal{D}}(\mathsf{op})_2]\!])),$$

where we write $Df$ for the semantic derivative of $f$ (see §5). We need to verify, respectively, that $([\![\mathsf{op}]\!],([\![\overrightarrow{\mathcal{D}}(\mathsf{op})_1]\!],[\![\overrightarrow{\mathcal{D}}(\mathsf{op})_2]\!]))$ and $([\![\mathsf{op}]\!],([\![\overleftarrow{\mathcal{D}}(\mathsf{op})_1]\!],[\![\overleftarrow{\mathcal{D}}(\mathsf{op})_2]\!]))$ respect the logical relations $P^f$ and $P^r$. This respecting of relations follows immediately from the chain rule for multivariate differentiation, as long as we have implemented our derivatives correctly for the basic operations $\mathsf{op}$:

$$[\![x;y\vdash D\mathsf{op}(x;y)]\!] = D[\![\mathsf{op}]\!]\qquad\text{and}\qquad [\![x;y\vdash (D\mathsf{op})^t(x;y)]\!] = (D[\![\mathsf{op}]\!])^t.$$

Writing $\mathbf{real}^{n_1,..,n_k}\overset{\text{def}}{=}\mathbf{real}^{n_1}*..*\mathbf{real}^{n_k}$ and $\mathbb{R}^{n_1,..,n_k}\overset{\text{def}}{=}\mathbb{R}^{n_1}\times..\times\mathbb{R}^{n_k}$, we compute

$$(\!|\mathbf{real}^{n_1,..,n_k}|\!)^f = ((\mathbb{R}^{n_1,..,n_k},(\mathbb{R}^{n_1,..,n_k},\underline{\mathbb{R}}^{n_1,..,n_k})),\{(f,(g,h)) \mid f = g, h = Df\})$$
$$(\!|\mathbf{real}^{n_1,..,n_k}|\!)^r = ((\mathbb{R}^{n_1,..,n_k},(\mathbb{R}^{n_1,..,n_k},\underline{\mathbb{R}}^{n_1,..,n_k})),\{(f,(g,h)) \mid f = g, h = (Df)^t\})$$

since derivatives of tuple-valued functions are computed component-wise. (In fact, the corresponding facts hold more generally for any first-order type, as an iterated product of $\mathbf{real}^n$.) Suppose that $(f,(g,h)) \in P^f_{\mathbf{real}^{n_1,\ldots,n_k}}$, i.e. $g = f$ and $h = Df$. Then, using the chain rule in the last step, we have

$$\begin{aligned}
&(f,(g,h));(\llbracket \mathsf{op} \rrbracket,(\llbracket \overrightarrow{\mathcal{D}}(\mathsf{op})_1 \rrbracket, \llbracket \overrightarrow{\mathcal{D}}(\mathsf{op})_2 \rrbracket)) = (f,(f,Df));(\llbracket \mathsf{op} \rrbracket,(\llbracket \mathsf{op} \rrbracket, \llbracket x; y \vdash D\mathsf{op}(x;y) \rrbracket))\\
&= (f,(f,Df));(\llbracket \mathsf{op} \rrbracket,(\llbracket \mathsf{op} \rrbracket, D\llbracket \mathsf{op} \rrbracket)) = (f;\llbracket \mathsf{op} \rrbracket,(f;\llbracket \mathsf{op} \rrbracket, x \mapsto r \mapsto D\llbracket \mathsf{op} \rrbracket(f(x))(Df(x)(r))))\\
&= (f;\llbracket \mathsf{op} \rrbracket,(f;\llbracket \mathsf{op} \rrbracket, D(f;\llbracket \mathsf{op} \rrbracket))) \in P^f_{\mathbf{real}^m}.
\end{aligned}$$

Similarly, if $(f,(g,h)) \in P^r_{\mathbf{real}^{n_1,\ldots,n_k}}$, then by the chain rule and linear algebra

$$\begin{aligned}
&(f,(g,h));(\llbracket \mathsf{op} \rrbracket,(\llbracket \overleftarrow{\mathcal{D}}(\mathsf{op})_1 \rrbracket, \llbracket \overleftarrow{\mathcal{D}}(\mathsf{op})_2 \rrbracket)) = (f,(f,(Df)^t));(\llbracket \mathsf{op} \rrbracket,(\llbracket \mathsf{op} \rrbracket, \llbracket x; y \vdash (D\mathsf{op})^t(x;y) \rrbracket)) =\\
&(f,(f,Df^t));(\llbracket \mathsf{op} \rrbracket,(\llbracket \mathsf{op} \rrbracket,(D\llbracket \mathsf{op} \rrbracket)^t)) = (f;\llbracket \mathsf{op} \rrbracket,(f;\llbracket \mathsf{op} \rrbracket, x \mapsto v \mapsto Df^t(x)(D\llbracket \mathsf{op} \rrbracket^t(f(x))(v)))) =\\
&(f;\llbracket \mathsf{op} \rrbracket,(f;\llbracket \mathsf{op} \rrbracket, x \mapsto v \mapsto (Df(x);D\llbracket \mathsf{op} \rrbracket(f(x)))^t(v))) = (f;\llbracket \mathsf{op} \rrbracket,(f;\llbracket \mathsf{op} \rrbracket,(D(f;\llbracket \mathsf{op} \rrbracket))^t)) \in P^r_{\mathbf{real}^m}.
\end{aligned}$$

Consequently, we obtain our Cartesian closed functors $\llparenthesis - \rrparenthesis^f$ and $\llparenthesis - \rrparenthesis^r$.

Further, observe that $\Sigma_{\llbracket - \rrbracket}\llbracket - \rrbracket(t_1,t_2) \stackrel{\text{def}}{=} (\llbracket t_1 \rrbracket, \llbracket t_2 \rrbracket)$ defines a Cartesian closed functor $\Sigma_{\llbracket - \rrbracket}\llbracket - \rrbracket : \Sigma_{\mathbf{CSyn}}\mathbf{LSyn} \to \Sigma_{\mathbf{Diff}}\mathbf{Diff_{CM}}$. Similarly, we get a Cartesian closed functor $\Sigma_{\llbracket - \rrbracket}\llbracket - \rrbracket^{op} : \Sigma_{\mathbf{CSyn}}\mathbf{LSyn}^{op} \to \Sigma_{\mathbf{Diff}}\mathbf{Diff_{CM}}^{op}$. As a consequence, the two squares below commute.

$$\begin{array}{ccc}
\mathbf{Syn} & \xrightarrow{(\mathrm{id},\overrightarrow{\mathcal{D}})} & \mathbf{Syn} \times \Sigma_{\mathbf{CSyn}}\mathbf{LSyn}\\
{\scriptstyle \llparenthesis - \rrparenthesis^f}\downarrow & & \downarrow{\scriptstyle \llbracket - \rrbracket \times \Sigma_{\llbracket - \rrbracket}\llbracket - \rrbracket}\\
\overrightarrow{\mathbf{SScone}} & \xrightarrow[\pi_1]{} & \mathbf{Diff} \times \Sigma_{\mathbf{Diff}}\mathbf{Diff_{CM}}
\end{array}
\qquad
\begin{array}{ccc}
\mathbf{Syn} & \xrightarrow{(\mathrm{id},\overleftarrow{\mathcal{D}})} & \mathbf{Syn} \times \Sigma_{\mathbf{CSyn}}\mathbf{LSyn}^{op}\\
{\scriptstyle \llparenthesis - \rrparenthesis^r}\downarrow & & \downarrow{\scriptstyle \llbracket - \rrbracket \times \Sigma_{\llbracket - \rrbracket}\llbracket - \rrbracket^{op}}\\
\overleftarrow{\mathbf{SScone}} & \xrightarrow[\pi_1]{} & \mathbf{Diff} \times \Sigma_{\mathbf{Diff}}\mathbf{Diff_{CM}}^{op}.
\end{array}$$

Indeed, going around the squares in both directions define Cartesian closed functors that agree on their action on $\mathbf{real}^n$ and all operations $\mathsf{op}$. So, by the universal property of $\mathbf{Syn}$, they must coincide. In particular, $(\llbracket t \rrbracket, (\llbracket \overrightarrow{\mathcal{D}}(t)_1 \rrbracket, \llbracket \overrightarrow{\mathcal{D}}(t)_2 \rrbracket))$ is a morphism in $\overrightarrow{\mathbf{SScone}}$ and therefore respects the logical relations $P^f$ for any well-typed term $t$ of the source language of §3. Similarly, $(\llbracket t \rrbracket, (\llbracket \overleftarrow{\mathcal{D}}(t)_1 \rrbracket, \llbracket \overleftarrow{\mathcal{D}}(t)_2 \rrbracket))$ is a morphism in $\overleftarrow{\mathbf{SScone}}$ and therefore respects the logical relations $P^r$.

Most of the work is now in place to show correctness of AD. We finish the proof below. To ease notation, we work with terms in a context with a single type. Doing so is not a restriction as our language has products, and the theorem holds for arbitrary terms between first-order types.

**Theorem 1 (Correctness of AD).** *For programs $x : \tau \vdash t : \sigma$ between first-order types $\tau$ and $\sigma$,*

$$\llbracket \overrightarrow{\mathcal{D}}(t)_1 \rrbracket = \llbracket t \rrbracket \qquad \llbracket \overrightarrow{\mathcal{D}}(t)_2 \rrbracket = D\llbracket t \rrbracket \qquad \llbracket \overleftarrow{\mathcal{D}}(t)_1 \rrbracket = \llbracket t \rrbracket \qquad \llbracket \overleftarrow{\mathcal{D}}(t)_2 \rrbracket = D\llbracket t \rrbracket^t,$$

*where we write $D$ and $(-)^t$ for the usual calculus derivative and matrix transpose.*

*Proof (sketch, see [36, Appx. B] for details).* To show that $\llbracket \overrightarrow{\mathcal{D}}(t)_1 \rrbracket(x) = \llbracket t \rrbracket(x)$ and $\llbracket \overrightarrow{\mathcal{D}}(t)_2 \rrbracket(x)(v) = D\llbracket t \rrbracket(x)(v)$, we choose a smooth curve $\gamma : \mathbb{R} \to \llbracket \tau \rrbracket$ such that $\gamma(0) = 0$ and $D\gamma(0)(1) = v$ and use that $t$ respects the logical relations $P^f$.

To show that $\llbracket \overleftarrow{\mathcal{D}}(t)_1 \rrbracket(x) = \llbracket t \rrbracket(x)$ and $\llbracket \overleftarrow{\mathcal{D}}(t)_2 \rrbracket(x)(v) = D\llbracket t \rrbracket(x)^t(v)$, we choose smooth curves $\gamma_i : \mathbb{R} \to \llbracket \tau \rrbracket$ such that $\gamma_i(0) = x$ and $\gamma_i(0)(1) = e_i$, for all standard basis vectors $e_i$ of $\llbracket \overleftarrow{\mathcal{D}}(\tau)_2 \rrbracket \cong \underline{\mathbb{R}}^N$. It now follows that $\llbracket \overleftarrow{\mathcal{D}}(t)_1 \rrbracket(x) = \llbracket t \rrbracket(x)$ and $e_i \bullet \llbracket \overleftarrow{\mathcal{D}}(t)_2 \rrbracket(x)(v) = e_i \bullet D\llbracket t \rrbracket(x)^t(v)$ as $t$ respects the logical relations $P^r$. $\square$

$$\frac{\Gamma\vdash t:\mathbf{Dom}(\mathsf{lop})\quad (\mathsf{lop}\in\mathsf{LOp}^m_{n_1,\ldots,n_k;n'_1,\ldots,n'_l})}{\Gamma\vdash\mathsf{lop}(t):\mathbf{LFun}(\mathbf{LDom}(\mathsf{lop}),\mathbf{real}^m)}\quad\frac{}{\Gamma\vdash\underline{0}_\tau:\tau}\quad\frac{\Gamma\vdash t:\tau\quad\Gamma\vdash s:\tau}{\Gamma\vdash t+_\tau s:\tau}$$

$$\frac{}{\Gamma\vdash\mathbf{lid}:\mathbf{LFun}(\tau,\tau)}\quad\frac{\Gamma\vdash t:\mathbf{LFun}(\tau,\sigma)\quad\Gamma\vdash s:\mathbf{LFun}(\sigma,\rho)}{\Gamma\vdash t;s:\mathbf{LFun}(\tau,\rho)}\quad\frac{\Gamma\vdash t:\mathbf{LFun}(\tau,\sigma)\quad\Gamma\vdash s:\tau}{\Gamma\vdash\mathbf{lapp}(t,s):\sigma}$$

$$\frac{\Gamma\vdash t:\tau\to\mathbf{LFun}(\sigma,\rho)}{\Gamma\vdash\mathbf{lswap}\,t:\mathbf{LFun}(\sigma,\tau\to\rho)}\quad\frac{\Gamma\vdash t:\tau}{\Gamma\vdash\mathbf{leval}_t:\mathbf{LFun}(\tau\to\sigma,\sigma)}$$

$$\frac{\Gamma\vdash t:\tau}{\Gamma\vdash\{(t,-)\}:\mathbf{LFun}(\sigma,\mathbf{Tens}(\tau,\sigma))}\quad\frac{\Gamma\vdash t:\tau\to\mathbf{LFun}(\sigma,\rho)}{\Gamma\vdash\mathbf{lcur}^{-1}t:\mathbf{LFun}(\mathbf{Tens}(\tau,\sigma),\rho)}\quad\frac{}{\Gamma\vdash\mathbf{lfst}:\mathbf{LFun}(\tau*\sigma,\tau)}$$

$$\frac{}{\Gamma\vdash\mathbf{lsnd}:\mathbf{LFun}(\tau*\sigma,\sigma)}\quad\frac{\Gamma\vdash t:\mathbf{LFun}(\tau,\sigma)\quad\Gamma\vdash s:\mathbf{LFun}(\tau,\rho)}{\Gamma\vdash\mathbf{lpair}(t,s):\mathbf{LFun}(\tau,\sigma*\rho)}$$

**Fig. 5.** Typing rules for the applied target language, to extend the source language.

### 9 Practical Relevance and Implementation

Popular functional languages, such as Haskell and O'Caml, do not natively support linear types. As such, the transformations described in this paper may seem hard to implement. However, as we summarize in this section (and detail in [36, Appx. C]), we can easily implement the limited linear types needed for the transformations as abstract data types by using merely a basic module system.

Specifically, we consider, as an alternative, applied target language for our transformations, the extension of the source language of §3 with the terms and types of Fig. 5. We can define a faithful translation $(-)^\dagger$ from our linear target language of §4 to this language: define $(!\tau\otimes\underline{\sigma})^\dagger\stackrel{\text{def}}{=}\mathbf{Tens}(\tau^\dagger,\underline{\sigma}^\dagger,)$, $(\underline{\tau}\multimap\underline{\sigma})^\dagger\stackrel{\text{def}}{=}\mathbf{LFun}(\underline{\tau}^\dagger,\underline{\sigma}^\dagger)$, $(\underline{\mathbf{real}}^n)^\dagger\stackrel{\text{def}}{=}\mathbf{real}^n$ and extend $(-)^\dagger$ structurally recursively, letting it preserve all other type formers. We then translate $(x_1:\tau,\ldots,x_n:\tau;y:\underline{\sigma}\vdash t:\underline{\rho})^\dagger\stackrel{\text{def}}{=}x_1:\tau^\dagger,\ldots,x_n:\tau^\dagger\vdash t^\dagger:(\underline{\sigma}\multimap\underline{\rho})^\dagger$ and $(x_1:\tau,\ldots,x_n:\tau\vdash t:\sigma)^\dagger\stackrel{\text{def}}{=}x_1:\tau^\dagger,\ldots,x_n:\tau^\dagger\vdash t^\dagger:\sigma^\dagger$. We believe an interested reader can fill in the details. This exhibits the linear target language as a sublanguage of the applied target language. The applied target language merely collapses the distinction between linear and Cartesian types and it adds the constructs $\mathbf{lapp}(t,s)$ for practical usability and to ensure that our adequacy result below is meaningful.

We can implement the API of Fig. 5 as a module that defines the abstract types $\mathbf{LFun}(\tau,\sigma)$, under the hood implemented as a plain function type $\tau\to\sigma$, and $\mathbf{Tens}(\tau,\sigma)$, which is implemented as lists of pairs $\mathbf{List}(\tau*\sigma)$. Then, the required terms of Fig. 5 can be implemented as follows, using standard idiom $[\,]$, $t::s$, $\mathbf{fold}\,op\,\mathbf{over}\,x\,\mathbf{in}\,t\,\mathbf{from}\,acc=init$ for empty lists, cons-ing, and folding:

$$\underline{0}_{\mathbf{1}}=\langle\rangle\quad t+_{\mathbf{1}}s=\langle\rangle\quad\underline{0}_{\underline{\tau}*\underline{\sigma}}=\langle\underline{0}_{\underline{\tau}},\underline{0}_{\underline{\sigma}}\rangle\quad t+_{\underline{\tau}*\underline{\sigma}}s=\langle\mathbf{fst}\,t+_{\underline{\tau}}\mathbf{fst}\,s,\mathbf{snd}\,t+_{\underline{\sigma}}\mathbf{snd}\,s\rangle$$

$$\underline{0}_{\tau\to\underline{\sigma}}=\lambda\_.\underline{0}_{\underline{\sigma}}\quad t+_{\tau\to\underline{\sigma}}s=\lambda x.t\,x+_{\underline{\sigma}}s\,x\quad\underline{0}_{\mathbf{LFun}(\tau,\underline{\sigma})}=\lambda\_.\underline{0}_{\underline{\sigma}}\quad t+_{\mathbf{LFun}(\tau,\underline{\sigma})}s=\lambda x.t\,x+_{\underline{\sigma}}s\,x$$

$$\underline{0}_{\mathbf{Tens}(\tau,\sigma)}\stackrel{\text{def}}{=}[\,]\quad t+_{\mathbf{Tens}(\tau,\sigma)}s\stackrel{\text{def}}{=}\mathbf{fold}\,x::acc\,\mathbf{over}\,x\,\mathbf{in}\,t\,\mathbf{from}\,acc=s$$

$$\mathbf{lid}\stackrel{\text{def}}{=}\lambda x.x\quad t;s\stackrel{\text{def}}{=}\lambda x.s\,(t\,x)\quad\mathbf{lapp}(t,s)\stackrel{\text{def}}{=}t\,s\quad\mathbf{lswap}\,t\stackrel{\text{def}}{=}\lambda x.\lambda y.t\,y\,x\quad\mathbf{leval}_t\stackrel{\text{def}}{=}\lambda x.x\,t$$

$$\{(t,-)\} \stackrel{\text{def}}{=} \lambda x.\langle t, x\rangle :: [\,] \quad \mathbf{lcur}^{-1} t \stackrel{\text{def}}{=} \lambda z.\mathbf{fold}\, t\, (\mathbf{fst}\, x)\, (\mathbf{snd}\, x) + acc\, \mathbf{over}\, x\, \mathbf{in}\, z\, \mathbf{from}\, acc = \underline{0}$$

$$\mathbf{lfst} \stackrel{\text{def}}{=} \lambda x.\mathbf{fst}\, x \quad \mathbf{lsnd} \stackrel{\text{def}}{=} \lambda x.\mathbf{snd}\, x \quad \mathbf{lpair}(t, s) \stackrel{\text{def}}{=} \lambda x.\langle t\, x, s\, x\rangle$$

Our denotational semantics extends to this applied target language and is adequate with respect to the operational semantics induced by the suggested implementation. Further, our correctness proofs of the induced source-code translations also transfer to this applied setting, and they can be usefully phrased as manual, extensible logical relations proofs. As an application, we can extend our source language with higher-order primitives, like $\mathbf{map} \in \mathbf{Syn}((\mathbf{real} \to \mathbf{real}){*}\mathbf{real}^n, \mathbf{real}^n)$ to "map" functions over the black-box arrays $\mathbf{real}^n$. Then, our proofs extend to show that their correct forward and reverse derivatives are

$$\overrightarrow{\mathcal{D}}(\mathbf{map})_1(f, v) \stackrel{\text{def}}{=} \mathbf{map}(f; \mathbf{fst}\,, v) \quad \overrightarrow{\mathcal{D}}(\mathbf{map})_2(f, v)(g, w) \stackrel{\text{def}}{=} \mathbf{map}\, g\, v + \mathbf{zipWith}(f; \mathbf{snd}\,)\, v\, w$$

$$\overleftarrow{\mathcal{D}}(\mathbf{map})_1(f, v) \stackrel{\text{def}}{=} \mathbf{map}(f; \mathbf{fst}\,, v) \quad \overleftarrow{\mathcal{D}}(\mathbf{map})_2(f, v)(w) \quad \stackrel{\text{def}}{=} \langle \mathbf{zip}\, v\, w, \mathbf{zipWith}\,(f; \mathbf{snd}\,)\, v\, w\rangle,$$

where we use the standard functional programming idiom **zip** and **zipWith**. Here, we can operate directly on the internal representations of $\mathbf{LFun}(\tau, \sigma)$ and $\mathbf{Tens}(\tau, \sigma)$, as the definitions of derivatives of primitives live inside our module.

### 10 Related and Future Work

**Related work** This work is closely related to [20], which introduced a similar semantic correctness proof for a version of forward-mode AD, using a subsconing construction. A major difference is that this paper also phrases and proves correctness of reverse-mode AD on a λ-calculus and relates reverse-mode to forward-mode AD. Using a syntactic logical relations proof instead, [5] also proves correctness of forward-mode AD. Again, it does not address reverse AD.

[11] proposes a similar construction to that of §6, and it relates it to the differential λ-calculus. This paper develops sophisticated axiomatics for semantic reverse differentiation. However, it neither relates the semantics to a source-code transformation, nor discusses differentiation of higher-order functions. Our construction of differentiation with a (biadditive) linear target language might remind the reader of differential linear logic [15]. In differential linear logic, (forward) differentiation is a first-class operation in a (biadditive) linear language. By contrast, in our treatment, differentiation is a meta-operation.

Importantly, [16] describes and implements what are essentially our source-code transformations, though they were restricted to first-order functions and scalars. [37] sketches an extension of the reverse-mode transformation to higher-order functions in essentially the same way as proposed in this paper. It does not motivate or derive the algorithm or show its correctness. Nevertheless, this short paper discusses important practical considerations for implementing the algorithm, and it discusses a dependently typed variant of the algorithm.

Next, there are various lines of work relating to correctness of reverse-mode AD that we consider less similar to our work. For example, [28] define and prove correct a formulation of reverse-mode AD on a higher-order language that depends on a non-standard operational semantics, essentially a form of symbolic execution. [2] does something similar for reverse-mode AD on a first-order language extended with conditionals and iteration. [8] defines an AD algorithm in a simply typed $\lambda$-calculus with linear negation (essentially, the continuation-based AD of [20]) and proves it correct using operational techniques. Further, they show that this algorithm corresponds to reverse-mode AD under a non-standard operational semantics (with the "linear factoring rule"). These formulations of reverse-mode AD all depend on non-standard run-times and fall into the category of "define-by-run" formulations of reverse-mode AD. Meanwhile, we are concerned with "define-then-run" formulations: source-code transformations producing differentiated code at compile-time, which can then be optimized during compilation with existing compiler tool-chains.

Finally, there is a long history of work on reverse-mode AD, though almost none of it applies the technique to higher-order functions. A notable exception is [31], which gives an impressive source-code transformation implementation of reverse AD in Scheme. While very efficient, this implementation crucially uses mutation. Moreover, the transformation is complex and correctness is not considered. More recently, [38] describes a much simpler implementation of a reverse AD code transformation, again very performant. However, the transformation is quite different from the one considered in this paper as it relies on a combination of delimited continuations and mutable state. Correctness is not considered, perhaps because of the semantic complexities introduced by impurity.

Our work adds to the existing literature by presenting (to our knowledge) the first principled and pure define-then-run reverse AD algorithm for a higher-order language, by arguing its practical applicability, and by proving semantic correctness of the algorithm.

**Future work** We plan to build a practical, verified AD library based on the methods introduced in this paper. This will involve calculating the derivative of many first- and higher-order primitives according to our method.

Next, we aim to extend our method to other expressive language features. We conjecture that the method extends to source languages with variant and inductive types as long as one makes the target language a linear dependent type theory [10,34]. Indeed, the dimension of (co)tangent spaces to a disjoint union of spaces depends on the choice of base point. The required colimits to interpret such types in $\Sigma_{\mathcal{C}}\mathcal{L}$ and $\Sigma_{\mathcal{C}}\mathcal{L}^{op}$ should exist by standard results about arrow and container categories [3]. We are hopeful that the method can also be made to apply to source languages with general recursion by calculating the derivative of fixpoint combinators similarly to our calculation for **map**. The correctness proof will then rely on a domain theoretic generalisation of our techniques [35].

**Acknowledgements** This project has received funding from the European Union's Horizon 2020 research and innovation programme under the Marie Skłodowska-Curie grant agreement No. 895827. We thank Michael Betancourt, Philip de Bruin, Bob Carpenter, Mathieu Huot, Danny de Jong, Ohad Kammar, Gabriele Keller, Pieter Knops, Curtis Chin Jen Sem, Amir Shaikhha, Tom Smeding, and Sam Staton for helpful discussions about automatic differentiation.
